# The Role of Protein and Free Amino Acids on Intake, Metabolism, and Gut Microbiome: A Comparison Between Breast-Fed and Formula-Fed Rhesus Monkey Infants

**DOI:** 10.3389/fped.2019.00563

**Published:** 2020-01-24

**Authors:** Xuan He, Jennie Sotelo-Orozco, Colin Rudolph, Bo Lönnerdal, Carolyn M. Slupsky

**Affiliations:** ^1^Department of Nutrition, University of California, Davis, Davis, CA, United States; ^2^Mead Johnson Nutrition, Evansville, IN, United States; ^3^Department of Food Science and Technology, University of California, Davis, Davis, CA, United States

**Keywords:** infant, formula-feeding, breastfeeding, low protein formula, metabolomics, microbiome

## Abstract

**Background:** Compared to breast-fed (BF), formula-fed (FF) infants exhibit more rapid weight gain, a different fecal microbial profile, as well as elevated serum insulin, insulin growth factor 1 (IGF-1), and branched chain amino acids (BCAAs). Since infant formula contains more protein and lower free amino acids than breast milk, it is thought that protein and/or free amino acids may be key factors that explain phenotypic differences between BF and FF infants.

**Methods:** Newborn rhesus monkeys (*Macaca mulatta*) were either exclusively BF or fed regular formula or reduced protein formula either supplemented or not with a mixture of amino acids. Longitudinal sampling and clinical evaluation were performed from birth to 16 weeks including anthropometric measurements, intake records, collection of blood for hematology, serum biochemistry, hormones, and metabolic profiling, collection of urine for metabolic profiling, and collection of feces for 16s rRNA fecal microbial community profiling.

**Results:** Reducing protein in infant formula profoundly suppressed intake, lowered weight gain and improved the FF-specific metabolic phenotype in the first month of age. This time-dependent change paralleled an improvement in serum insulin. All lower protein FF groups showed reduced protein catabolism with lower levels of blood urea nitrogen (BUN), urea, ammonia, albumin, creatinine, as well as lower excretion of creatinine in urine compared to infants fed regular formula. Levels of fecal microbes (*Bifidobacterium* and *Ruminococcus* from the Ruminococcaceae family), that are known to have varying ability to utilize complex carbohydrates, also increased with protein reduction. Adding free amino acids to infant formula did not alter milk intake or fecal microbial composition, but did significantly increase urinary excretion of amino acids and nitrogen-containing metabolites. However, despite the lower protein intake, these infants still exhibited a distinct FF-specific metabolic phenotype characterized by accelerated weight gain, higher levels of insulin and C-peptide as well as elevated amino acids including BCAA, lysine, methionine, threonine and asparagine.

**Conclusions:** Reducing protein and adding free amino acids to infant formula resulted in growth and metabolic performance of infants that were more similar to BF infants, but was insufficient to reverse the FF-specific accelerated growth and insulin-inducing high BCAA phenotype.

## Introduction

It is well-established that breast-fed (BF) infants exhibit different metabolic outcomes compared to formula-fed (FF) infants. This difference during early development is believed to influence the likelihood of developing health problems later in life such as overweight/obesity and diabetes (1–3). Although the detailed mechanism has not been fully elucidated, disruption of early-life gut microbiota may precede the development of obesity during childhood (4–6). In addition, the high protein content in infant formula has been shown to be a factor that is responsible for stimulation of higher insulin and insulin growth factor 1 (IGF-1) leading to rapid weight gain, while disfavoring the use of fat via lipolysis [the “early protein hypothesis” (7)].

Despite the differences in nutrient composition between breast milk and infant formula, bottle-feeding is recognized as a parent-led process that may lead to habitual overfeeding. FF infants tend to consume significantly greater volumes than BF infants (8, 9). However, it is not completely understood if this is due to feeding mode alone, the different nutrient composition between infant formula and human milk, or if human milk plays a role in how infants regulate intake. Non-human primates have been recognized as a valuable model for controlled dietary studies due to their similarity to humans with regard to neurobehavioral and metabolic development. Rhesus infants can be fed directly from birth *ad libitum* from a bottle resulting in demand-driven, self-regulating feeding. An advantage of this is that it allows for control of external feeding cues.

While free amino acids comprise approximately 5% of the total amino acid content in human milk (10), they are significantly lower in infant formula. Free amino acids in human milk provide for rapid absorption and utilization compared to protein-derived amino acids, and may lead to a different response of BF infants compared to FF infants. For example, glutamate, which is the free amino acid with highest concentration in breast milk (11), was shown to promote satiation and satiety as well as reduce total intake volume and energy consumed when added to infant formula (12). Thus, the presence of amino acids, in addition to the amount of protein in infant formulas may impact metabolic response. However, whether there is an interplay between the addition of free amino acids, intake and overall metabolism still remains to be investigated.

In this study, we used the rhesus monkey as a model to evaluate the physiological response and metabolic consequences of providing a formula with protein content that is slightly lower than the level present in rhesus monkey milk. We further tested whether reducing the protein level and incorporating free amino acids in the formula would decelerate weight gain, provide better support for self-regulation of energy intake, and result in a more similar physiological and metabolic response when compared to a BF reference group. We hypothesized that as the amount of protein and free amino acids in infant formula were modified to become more similar to the mother's milk, phenotypic improvement and a difference in fecal microbial profile would be observed. To investigate the time-dependent response after modulating protein and free amino acids composition in a cow milk-based infant formula on infant metabolism and gut microbiota, longitudinal sampling and clinical evaluation of 30 infant rhesus monkeys were performed from birth to 16 weeks of age. The current study compared the comprehensive metabolic implications of formula- and breast-feeding using an amino acid analyzer (AAA) and nuclear magnetic resonance (NMR) spectroscopy to characterize metabolic fingerprints from serum and urine, in combination with anthropometric measurements, intake records, serum hematology and biochemistry, metabolic hormones, and 16s rRNA fecal microbial community profiling.

## Materials and Methods

### Animals and Diets

Newborn rhesus monkeys (*Macaca mulatta*) in this study were randomly assigned to consume either mother's milk or one of four types of infant formula (regular formula, reduced protein formula, with or without addition of free amino acids that included alanine, glutamate, glutamine, taurine) from birth until 4 months of age (*n* = 6 female monkeys per dietary group). All monkeys were under constant care of nursery and vivarium staff. BF rhesus infants were exclusively breast-fed by their mothers and maintained outdoors. FF rhesus infants were housed individually in polycarbonate isolates with a surrogate mother (a terrycloth dummy) for the first month of life, and then matched with another monkey from the same group and housed in pairs for the reminder of the study. Throughout the study, infant monkeys were only separated briefly from their mother or their pair for sample collection.

After birth, the FF rhesus infants were hand-fed using a nursing bottle every 2 h until 5 days of age, where they progressed to use self-feeder for *ad libitum* access to the study formula. Animal care staff were blinded to the formula. Self-feeding training started the day after birth by placing the rhesus infants next to the feeder nipple with their surrogate mother and gently held in place while they self-fed. FF infants were not offered any monkey chow during the study. Fruits (banana and apple) were given in limited amounts after 3 months to allow them to explore and develop interest in novel foods. After 4 months of this study, all monkeys were returned to the colony. The study was conducted at California National Primate Research Center (CNPRC) in accordance with Department of Agriculture Animal Welfare Act. The study protocol was approved by University of California, Davis, Institutional Animal Care and Use Committee.

Experimental cow-milk infant formula was produced by Mead Johnson Nutrition (Evansville, IN, USA). Both regular and reduced protein formula contained 80% whey, 20% casein, 5.4 g of fat per 100 kcal. Regular formula contained 2.1 g protein and 11.1 g carbohydrate per 100 kcal. Reduced protein formula contained 1.8 g protein and 11.4 g carbohydrate per 100 kcal. Four amino acids were added to either the regular formula or reduced protein formula, to reach a target of 23 mg glutamate, 8.6 mg glutamine, 2.6 mg alanine, and 7.3 mg taurine per 100 kcal. Infant formula was prepared fresh by mixing 134 g of dry formula with 897 ml of water to make 1 L of formula to achieve final concentrations as shown in SI Table 1. Since the amount of added free amino acids was relatively low, the difference in the overall protein content (as evaluated by nitrogen content) was <2%.

### Sample Collection

**Figure 1:**
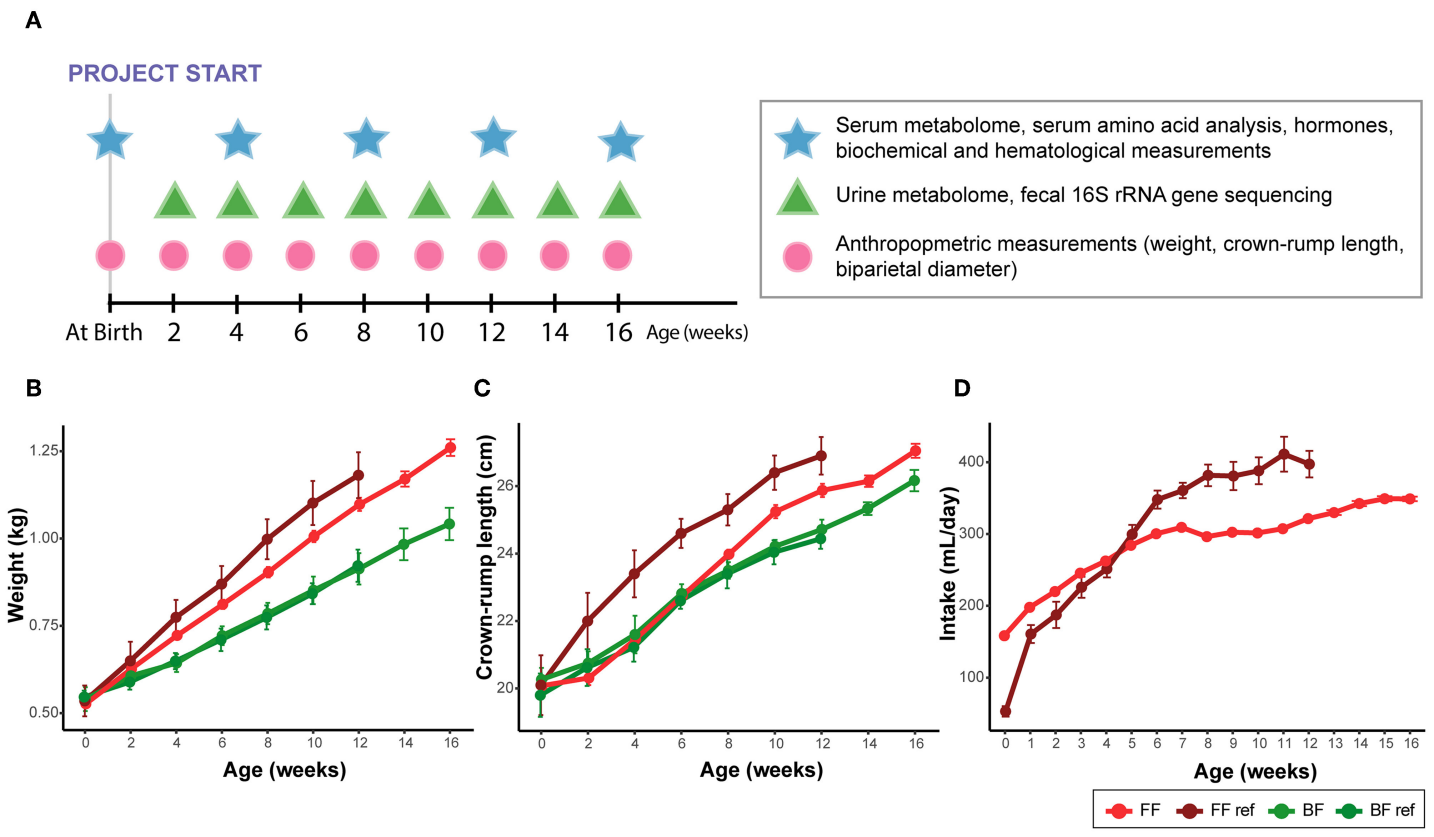
**(A)** Summary of sample collection times. **(B)** Weight, **(C)** crown-rump length, and **(D)** formula intake of infant monkeys from birth to age of 16 weeks. Data were collected from breast-fed (BF) and pooled among all formula-fed (FF) rhesus infants from the current study and from the previous work [BF ref, FF ref (14)]. The amount of milk obtained from the exclusively breast-fed rhesus monkeys could not be recorded. Data are presented as mean ± SEM.

Weight (g), crown-rump length (cm) and biparietal diameter (mm) were recorded at birth and every 2 weeks thereafter. Intake (mL/day) was recorded daily for the FF groups from birth to the end of the study. According to clinical practice and infant care standards, all rhesus infants were fed frequently and on demand, therefore, they were not specifically fasted prior to blood collection. Blood samples (1–3 mL) were drawn monthly via femoral venipuncture to a serum separator tube while hand-restrained. Samples were allowed to clot at room temperature for 30 min followed by centrifugation. Urine and fecal samples were collected biweekly (typically for <20 min but up to 4 h) using a specially designed metabolic unit as previously described (13). A summary of sample collection times is provided in Figure 1A.

### ^1^H NMR Metabolomics Analysis of Serum and Urine

Sample preparation and data acquisition were virtually identical to our previous monkey work (14). In brief, serum samples were filtered through a 4 kDa (3,000 MW) cut-off centrifugal filter (Amicon, Millipore, Billerica, MA) to remove macromolecules. The filtrate and urine were then prepared for analysis by addition of an internal standard containing 3-(trimethylsilyl)-1-propanesulfonic acid-d6 (DSS-d6) and 0.2% NaN3 in 99.8% D_2_O. The pH of each sample was adjusted to 6.8 ± 0.1 by adding small amounts of NaOH or HCl. After loading samples into NMR tubes, samples were run on a Bruker 600 MHz NMR spectrometer and acquired using the NOESY-presaturation pulse sequence (noespyr) at 25°C with water saturation during the 2.5 s prescan delay, a mixing time of 100 ms, 12 ppm sweepwidth, 2.5 s acquisition time, 8 dummy scans and 32 transients.

Quantified metabolites were derived from targeted profiling analysis using Chenomx NMRSuite (Chenomx Inc., Edmonton, Canada). All FIDs were multiplied by an exponential weighting function corresponding to a line broadening of 0.5 Hz. Spectra were manually phased, baseline-corrected and referenced to the DSS-d6 singlet at δ 0 ppm. All compounds in the database have been verified against known concentrations of reference NMR spectra of the pure compounds and have been shown to be reproducible and accurate (15). Eight urine samples shown to have high levels of acetate, butyrate, and propionate were suspected to be contaminated with feces, and therefore were removed from statistical analysis.

### Targeted Amino Acid Analysis of Serum

To prepare deproteinized extracts, 200 μL of serum samples were acidified with 50 μL sulfosalicylic acid. Intact proteins were removed after centrifugation and the supernatant from each sample was mixed with lithium diluent spiked with *S*-2-aminoethyl-L-cysteine. Samples were injected into an automated AAA (L-8900 Hitachi High-Technologies Corporation, Tokyo, Japan) equipped with ion-exchange chromatography using lithium citrate buffer. Each metabolite was detected spectrophotometrically after post-column reaction with ninhydrin reagent. Amino acid standards were intercalated with each sample and analyzed with a method developed by the Molecular Structure Facility (a part of UC Davis Proteome Core facility).

### Hematology Measurements, Serum Biochemistry, and Hormone Assays

Samples of whole blood were collected for red blood cell (RBC) count, white blood cell (WBC) count, hemoglobin, hematocrit, mean corpuscular volume (MCV), mean corpuscular hemoglobin (MCH), mean corpuscular hemoglobin platelet counts (MCHC), plasma protein, neutrophils, monocytes (%), lymphocytes (%), eosinophils (%), basophils (%), platelets, plasma color, fibrinogen, erythrocyte morphology. Hemoglobin, hematocrit, WBCs, and RBCs were quantified with an automated electronic cell counter (Baker 9010 Analyzer; Serono-Baker, Allentown, PA). Hematological measurements and smear evaluations were performed at the CNRPC clinical laboratory with standard quality assurance procedures.

The standardized clinical biochemistry panel has been modified for use with rhesus monkeys and was performed at the UC Davis Veterinary Medical Teaching Hospital's clinical laboratory using an automated analyzer (Hitachi 917, Roche Biomedical, Indianapolis, IN) with standard quality assurance procedures. Measurements included sodium, potassium, chloride, total carbon dioxide (TCO2), anion gap, inorganic phosphorous, calcium, blood urea nitrogen (BUN), creatinine, glucose, total protein, albumin, alanine aminotransferase (ALT), aspartate aminotransferase (AST), creatine phosphokinase (CPK), alkaline phosphatase (ALK PHOS), gamma glutamyl transferase (GGT), lactate dehydrogenase (LDH), triglyceride, total cholesterol, and direct bilirubin.

Serum C-peptide, GIP, GLP-1, insulin, leptin, MCP-1, pancreatic polypeptide and PYY (total) were measured using a 96-well multiplex hormone magnetic bead panel that is specifically designed for non-human primates (Cat# NHPMHMAG-45, Milliplex Analyst, Millipore) according to the manufacturer's instructions.

### Fecal Microbial 16s rRNA Gene Sequencing

DNA was extracted from monkey stool samples collected at the second week and every 2 weeks thereafter until 16 weeks of age according to the protocol used in our previous rhesus monkey work (14). Briefly, fecal samples were washed with ice-cold PBS, followed by various of steps involving chemical lysis (Lysis buffer), heat treatment, physical lysis (bead beating), and use of QIAamp DNA Stool Mini Kit (Qiagen, Valencia, CA). Fecal DNA samples were amplified using the primer pair F515 and R806 against the Variable region 4 (V4) of bacterial 16S ribosomal RNA genes. Purified DNA libraries were submitted to the UC Davis Genome Center DNA Technologies Core for 250 bp paired-end sequencing on the Illumina Miseq platform. The Paired-end sequences were analyzed in Quantitative Insights Into Microbial Ecology (QIIME) pipeline v.1.9.0 (16). A closed-reference OTU picking procedure was used against the most current Greengenes core database (“gg_13_8_otus”) (17). Differences in microbial community structures were explored using log-transformed weighted UniFrac distances followed by Principal Coordinate Analysis (PCoA). Differential abundance was evaluated using ANCOM (18) followed by FDR correction.

### Statistical Analysis

To approximate normality, all data (weight, crown-rump length, biparietal diameter, metabolite concentrations) were log 10 transformed. Upon identification of significant variables, univariate statistical analyses were performed to confirm between-group differences. To evaluate the treatment effect across the entire dataset, both repeated measures ANOVAs (after removal of the measurements at birth, if measured) and repeated measures ANCOVAs (with cofactor as measurement at birth) were compared (*lme* function in nlme package, R). For each variable, repeated measures ANCOVA was used only in the situation that the effect of baseline (measurements at birth, if measured) was significant after model comparison using ANOVA.

To investigate the effect of protein content and addition of free amino acids on growth outcome, serum biochemistry, hormone, hematology, metabolome and fecal microbiome data, subsequent 3-way repeated measures ANOVA or ANCOVA were performed on all the FF groups with main effects as protein level, addition of free amino acid and time. The differences in month 1 were evaluated using data collected at both 2 and 4 weeks (urine data) and data at 4 weeks of age alone (serum data) using multiple independent *t*-tests or 2-way ANOVA follow by *post-hoc* Tukey. Pearson's product-moment correlation was used to compare the difference between circulating C-peptide, insulin and branched chain amino acids (BCAAs). The significance of correlation was evaluated using *t*-tests under the null hypothesis that the correlation was zero. FDR adjustment was used to correct for the multiple pairwise comparisons.

HOMA-IR was calculated as the product of glucose (mmol/L) × insulin (mIU/L) /22.5. QUICKI was calculated as the product of 1/[log(I) + log(G)] where I is insulin (μU/mL) and G is fasting glucose (mg/dL).

## Results

### Rhesus Infants Consuming a Formula With a Protein Content Slightly Lower Than Rhesus Milk Still Revealed Higher Weight Gain, Insulin, and Elevated Circulating Amino Acids

We previously reported that when feeding a formula with higher protein content than rhesus milk, FF infants had an accelerated growth trajectory in comparison to their BF counterparts (14). In the present study, when feeding formulas with a slightly lower protein content than that of rhesus milk, all the FF infants exhibited moderate weight gain compared with the FF group from our previous work, but still showed faster weight gain compared with their BF counterparts (Figure 1B, pairwise comparison against all formula-fed groups, repeated measures ANCOVA, all individual *p* < 0.03); however, the differences in crown-rump length (Figure 1C) and biparietal diameter were not significant. The difference observed in the present study compared to the FF group from our previous work may be due to a lower intake of formula (Figure 1D). Nutrient composition of the diets is presented in SI Table 1.

**Figure 2:**
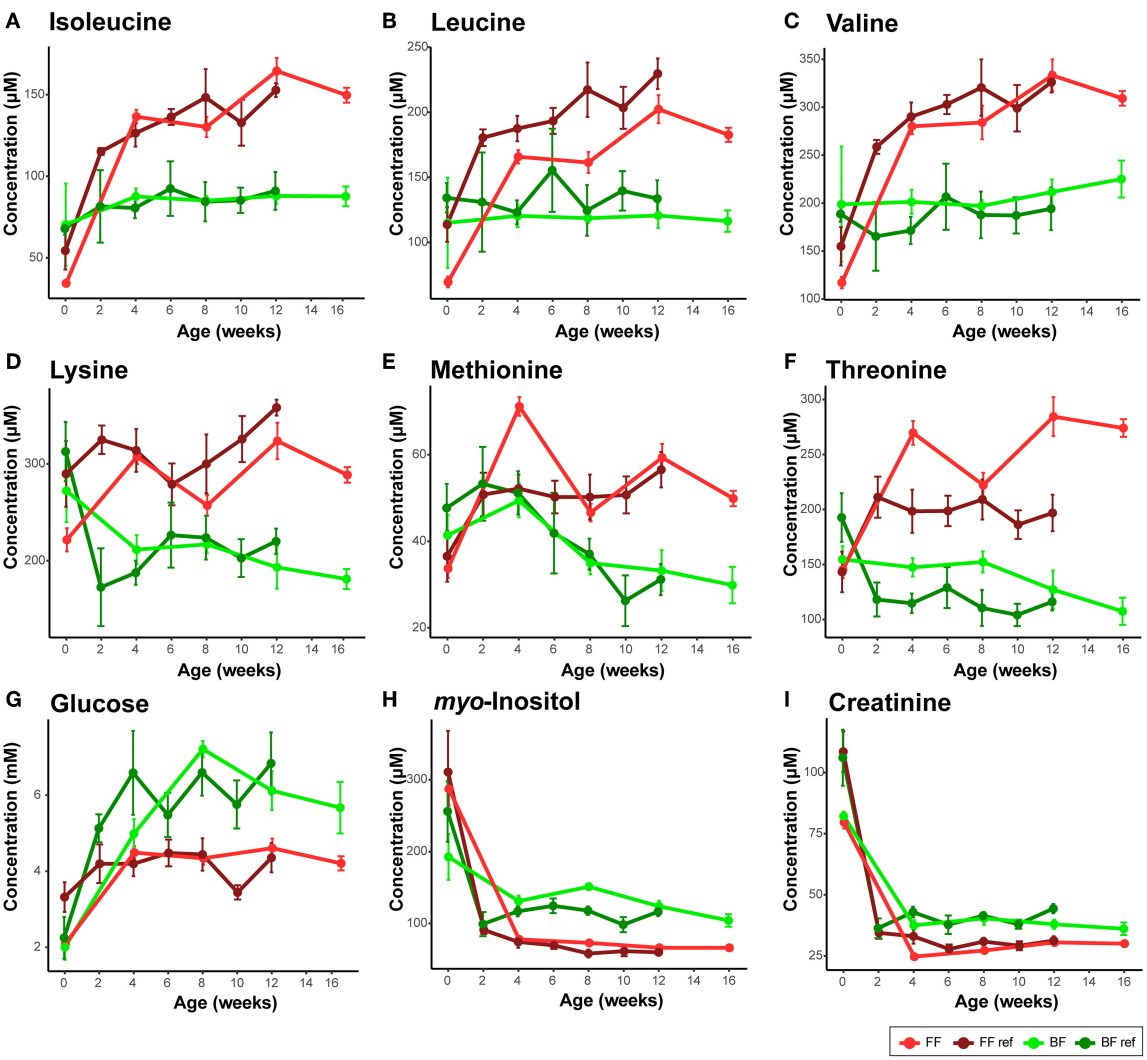
Comparison of serum metabolites that are consistent between the breast-fed (BF) and formula-fed (FF) rhesus infants from the current study and the breast-fed reference (BF ref) and formula-fed reference (FF ref) from previous work (14). Serum metabolites including **(A)** isoleucine, **(B)** leucine, **(C)** valine, **(D)** lysine, **(E)** methionine, **(F)** threonine, **(G)** glucose, **(H)** *myo*-inositol, **(I)** creatinine were measured from birth to 16 weeks of age in the current study. Data are presented as mean ± SEM.

In human infants, we and others have reported higher circulating nitrogenous waste products (urea/BUN) in FF compared to BF infants (19–22). Our previous work on infant rhesus macques did not find a significant difference in serum and urinary urea between infants who were breast-fed and those fed infant formula designed for human infants (14), which may be due to a higher dietary protein requirement of rhesus infants than human infants (23). In the present study, as formula protein was reduced to slightly lower than typically observed in rhesus milk, we observed significantly lower serum urea and ammonia in infants consuming formula compared to their BF counterparts (Repeated measures ANCOVA, SI Figure 1). Serum creatinine, another nitrogenous compound that is not different between BF and FF human infants, was consistently lower in the FF rhesus infants (Figure 2I, confirmed using both ^1^H NMR-based metabolomics analysis and clinical biochemical assays, SI Figure 2). We further quantified the urinary metabolites and observed a significantly lower creatinine level in these FF infants, which was not observed in our previous rhesus infant study (SI Figure 3).

Through assessment of a panel of clinical biomarkers, measurements of ALT, AST, and GGT that were previously reported to be higher in human BF infants (22) were either not different or higher levels were found in FF rhesus infants (summarized in Table 1, SI Figure 5). A panel of biochemical and hematological measurements revealed that serum albumin, a marker of undernutrition, was significantly lower in these FF rhesus infants. This lower level of albumin was coupled with lowered anion gap in these FF infants (SI Figure 5). Significantly lower hemoglobin, hematocrit and MCHC values were also observed (SI Figure 7), but were not significantly different from BF infants in our previous monkey study (14).

**Table 1:**
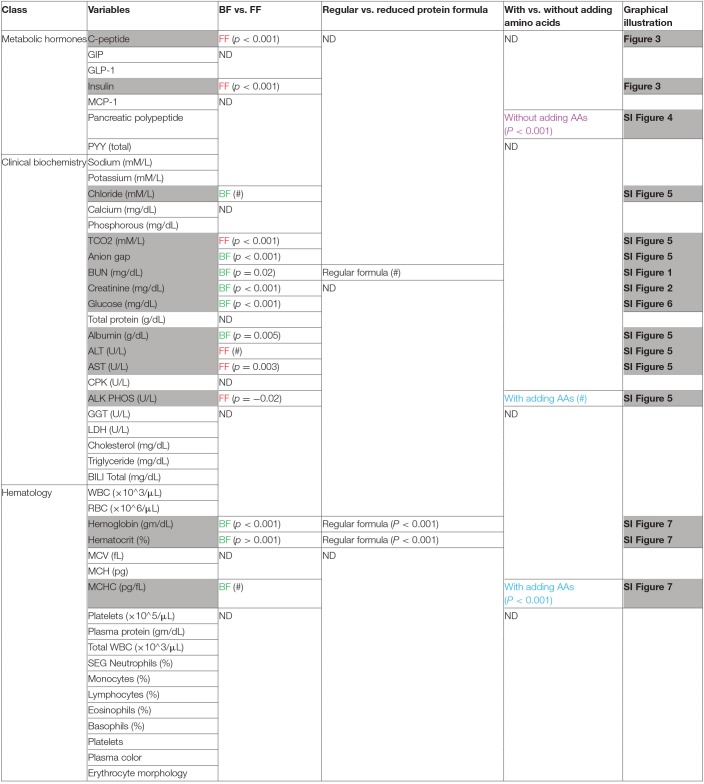
Metabolic hormones, biochemical measurements, hematological measurements.

*FF, higher in the formula-fed group; BF, higher in the breast-fed group; Regular formula, higher in the regular formula than reduced protein formula; With adding AAs, higher in formula groups with addition of free amino acids; Without adding AAs, higher in formula groups without addition of free AAs*.

*Significant difference was evaluated independently using repeated measures ANCOVA at p <0.05 after FDR correction. (#) p <0.05 before FDR correction. ND, not significantly different*.

Importantly, through ^1^H NMR-based metabolomics analysis, we found that when feeding the formula containing a level of protein approaching the minimum protein requirement of developing rhesus infants, circulating levels of BCAAs (leucine, isoleucine, and valine), lysine, methionine, threonine and asparagine reported to be higher in FF human infants and FF rhesus infants (from our previous work) were still significantly higher in the rhesus infants fed the current study formulas (Figure 2, Table 2), suggesting that reducing protein content in formula alone cannot completely reverse the formula feeding-specific metabolic phenotype on serum amino acid levels. Higher levels of circulating serine and phenylalanine were found in our previous study on FF rhesus infants but were not significantly different in the current study (SI Figure 13). Targeted amino acid analysis, performed in parallel with NMR-based metabolomics analysis, revealed similar results (summarized in Table 2).

**Table 2:** Breast and formula feeding- specific metabolite markers in serum.

**Class**	**Serum metabolites**	**Human infant reference ^[a]^**	**Human infant reference ^[b]^**	**Rhesus infant reference ^[c]^**	**Current study**	**Confirmed using amino acid analyzer**	**Graphical illustration**
Essential amino acids	Leucine	FF	FF	FF	FF	Yes	SI Figure 8
	Isoleucine	FF	FF	FF	FF	Yes	SI Figure 8
	Valine	FF	FF	FF	FF	Yes	SI Figure 8
	Lysine	FF	FF	FF	FF	Yes	SI Figure 9
	Phenylalanine			FF	ND	Yes	
	Methionine	FF	FF	FF	FF	Yes	SI Figure 9
	Threonine	FF	FF	FF	FF	Yes	SI Figure 9
	Tryptophan				FF	No^*^	
	Histidine				ND	Yes	
Non-essential amino acids	Alanine			FF	FF	Yes	SI Figure 10
	Arginine			FF	ND	Yes	
	Asparagine		FF	FF	FF	Yes	SI Figure 10
	Aspartate			FF	ND	Yes	
	Glutamate		BF	FF	ND	Yes	
	Glutamine			BF	ND	Yes	
	Serine		BF	FF	ND	Yes	
	Taurine			FF	ND	Yes	
	Tyrosine	FF	FF		ND	Yes	
	Proline	FF			ND	Yes	
	Creatine	FF	FF		ND	Yes	
	Glycin				ND	Yes	
	Ornithine				ND	Yes	
Amino acid derivatives	Hydroxyproline				BF	Yes	SI Figure 10
	Dimethylamine				BF		
	Creatinine			BF	BF		SI Figure 2
Sugars	Glucose			BF	BF		SI Figure 6
	Galactose			FF	FF		SI Figure 6
	*myo*-inositol		BF	BF	BF		SI Figure 6
Energy metabolism	Pyruvate			BF	BF		SI Figure 11
	Citrate		BF	BF	ND		
	Succinate		BF	BF	BF		SI Figure 11
	Fumarate		BF	BF	BF		SI Figure 11
	Lactate				BF		SI Figure 11
	Malate				BF		SI Figure 11
	Acetylcarnitine		BF		BF		
Ketones	3-hydroxybutyrate		BF		BF		SI Figure 12
	Acetoacetate		BF	BF	BF		SI Figure 12
Others	Allantoin			FF	ND		
	Urea	FF	FF	ND	FF		SI Figure 1

*Significant difference was evaluated independently using repeated measures 2-way ANOVA or ANCOVA at p < 0.05 after FDR correction. FF, higher in the formula-fed group; BF, higher in the breast-fed group; ND, no significant difference*.

[a] *Data from NMR metabolomics work extracted from a human study at approximately 180 min post-meal (21)*.

[b] *Data from NMR metabolomics work extracted from a human study at approximately 165 min post-meal with either no or little complementary food consumed (20)*.

[c] *Data extracted from our previous rhesus monkey study (14). Formula composition from this study is also summarized in SI Table 1*.

[a–c] Sample preparation, spectral acquisition and compound quantification followed the same protocol as the current study.

* *In the current study, serum tryptophan was significantly higher in the FF group. However, the protein precipitation method used in preparation of AAA released higher levels of protein-bound tryptophan compared to the ultrafiltration method used in the NMR sample preparation that captured only free tryptophan. The trend is in agreement with our previous work that also reported on samples extracted using protein precipitation or ultrafiltration (14)*.

**Figure 3:**
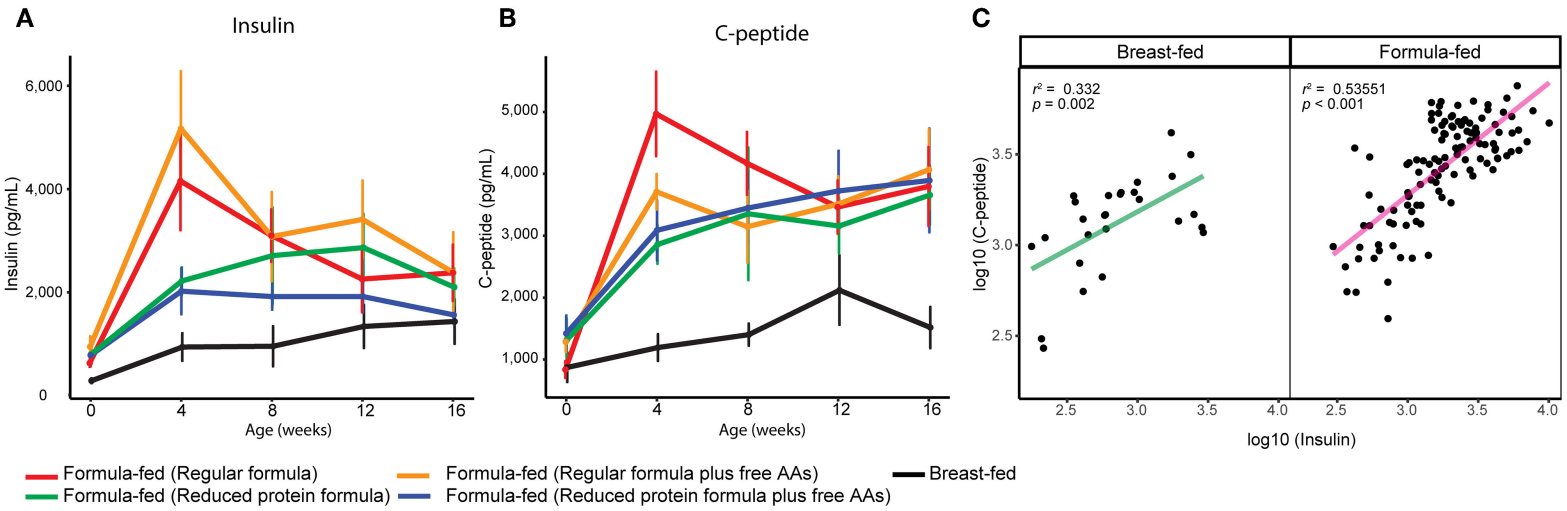
Serum **(A)** insulin and **(B)** C-peptide of breast-fed and formula-fed rhesus infants from birth to 16 weeks of age. Data are presented as mean ± SEM. **(C)** Scatter plot demonstrates a positive correlation between serum measurements of insulin and c-peptide.

Serum insulin was elevated in rhesus infants fed formula with a protein level higher than rhesus milk (14), and was still significantly higher in FF rhesus monkeys fed the current formula (repeated measures ANCOVA, Figure 3A). Serum C-peptide was also significantly higher in these FF infants and correlated well with serum insulin levels (repeated measures ANCOVA, Figures 3B,C). The higher levels of serum insulin and C-peptide are strongly correlated with higher serum isoleucine, leucine, valine, methionine, and threonine (Pearson correlation *r* > 0.4, *p* < 0.001 after FDR adjustment), supporting the regulatory role of these amino acids on insulin and C-peptide secretion (24). We have previously observed this positive relationship between BCAAs and insulin in human infants, suggesting this observation is translational to human infants (21).

Some breast feeding-specific markers were consistently altered. In agreement with observations in BF human infants, BF rhesus infants also exhibited higher levels of circulating *myo*-inositol, succinate, fumarate, and ketone bodies (summarized in Table 2). Circulating glucose and pyruvate were consistently higher in BF rhesus infants, but were not observed to be different between BF and FF human infants (Figure 2, Table 2). The differences in glucose were confirmed using clinical biochemical assays (SI Figure 6).

### Reducing the Protein Content of Infant Formula Suppresses Intake, Lowers Weight Gain, and Improves the Formula-Fed Specific Metabolic Phenotype

**Figure 4:**
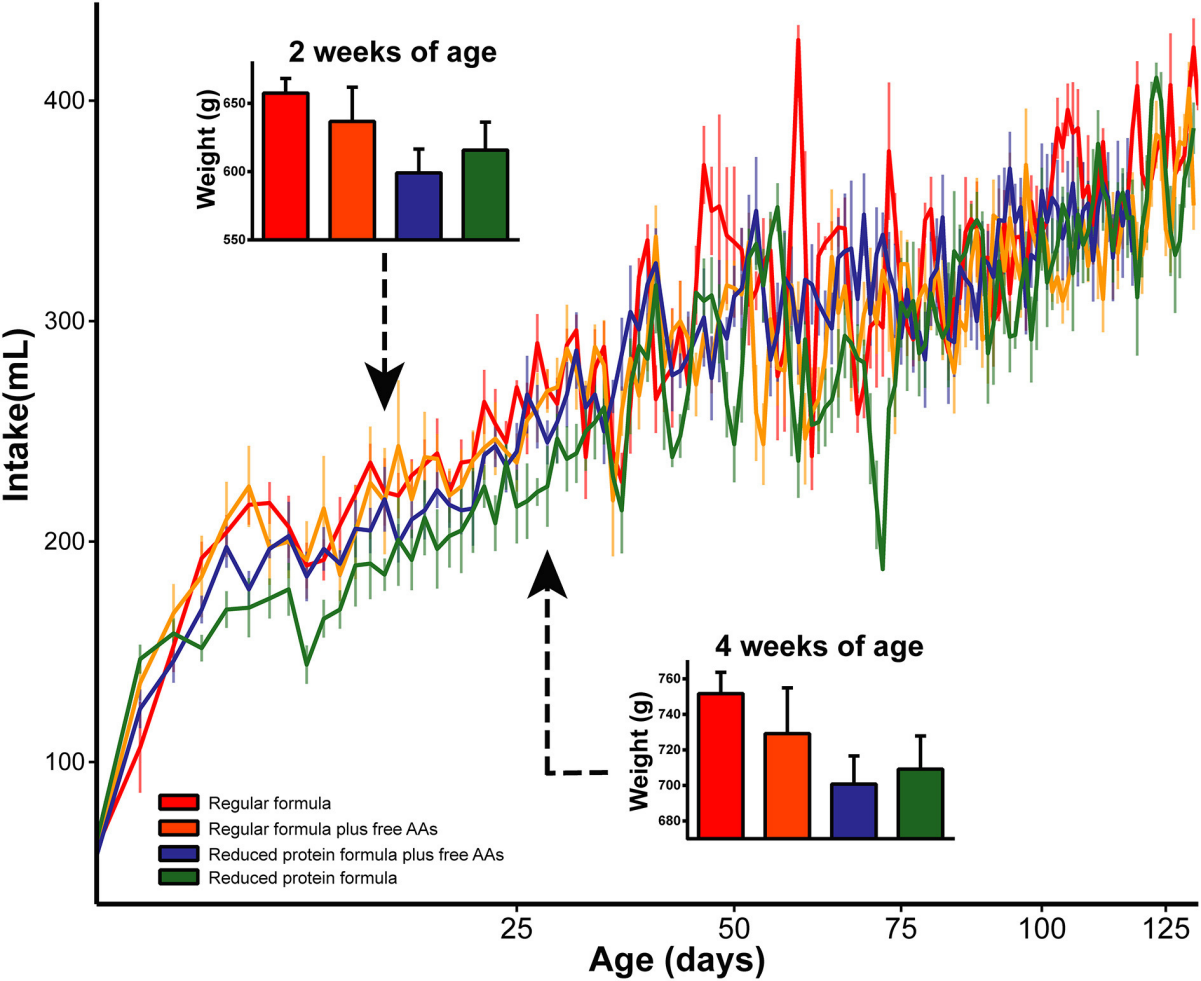
Daily intake was significantly increased by increasing the amount of protein in infant formula. As a result, weights at 2 and 4 weeks of age were significantly increased by the elevated protein content in infant formula. Data are presented as mean ± SEM.

To determine the metabolic impact of feeding a reduced protein formula, differences between infants fed regular and reduced protein formula were evaluated. Overall, the reduced protein formula had a protein composition identical to regular formula, but 14.3% less of each amino acid. Correspondingly, rhesus infants receiving the reduced protein formula showed 10–20% lower protein intake throughout the study compared to those fed regular formula (SI Figure 14). The lower protein content moderately decreased overall formula intake (*p* = 0.03, repeated measures ANCOVA), and this effect on appetite was most profound in the first month. Correspondingly, feeding the reduced protein formula significantly decreased weight gain measured at 2 and 4 weeks of age (*p* < 0.05, 3-way ANCOVA), but not at later time points (Figure 4).

**Figure 5:**
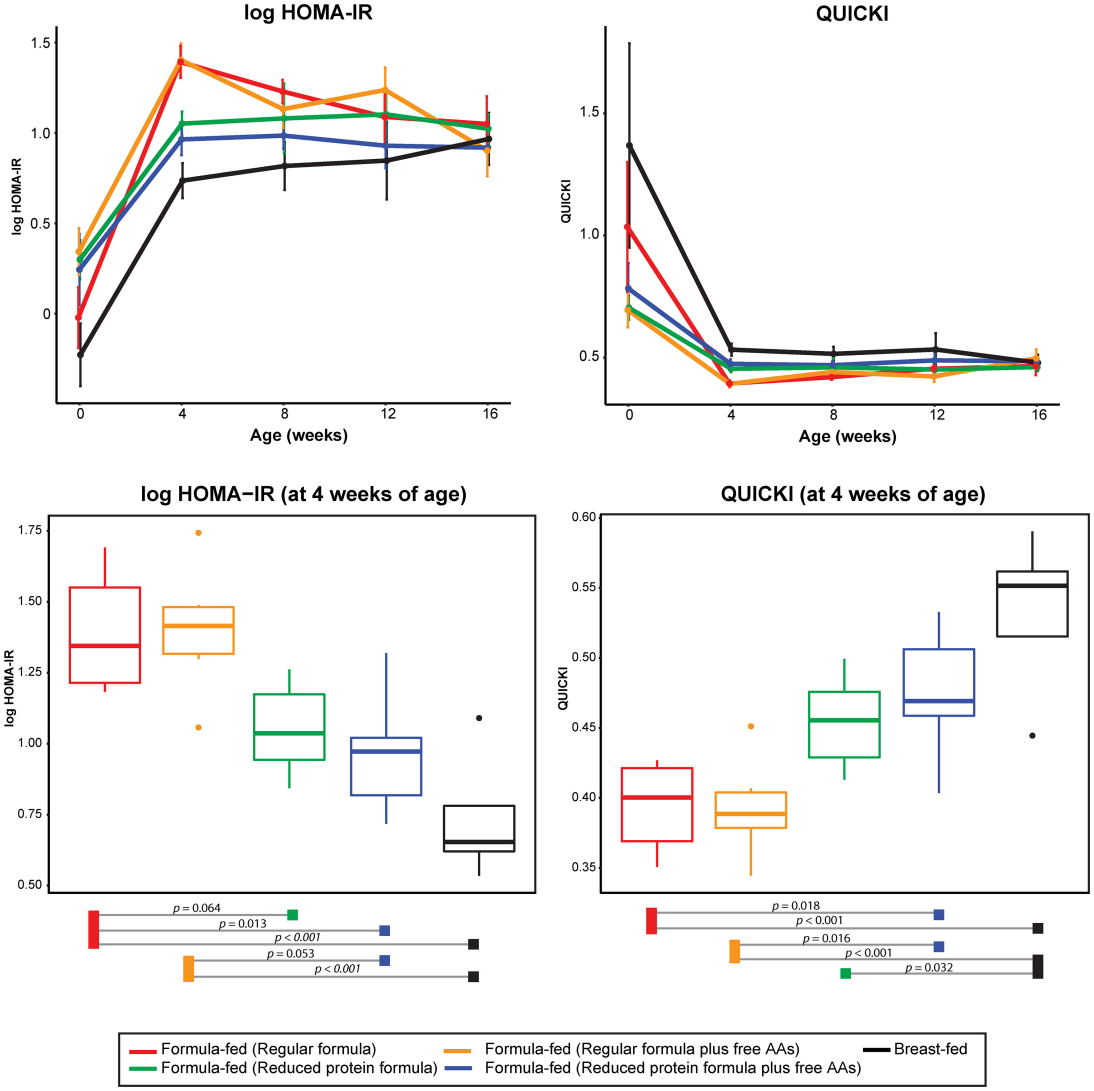
Change of log transformed HOMA-IR and QUICKI level over time and at 4 weeks of age. The statistical difference at 4 weeks of age was evaluated using ANOVA follow by *post-hoc* Tukey. Data are presented as mean ± SEM.

In parallel with the changes in formula intake and weight gain, at 4 weeks of age lower levels of circulating insulin and C-peptide as well as lower insulin resistance (homeostasis model assessment for insulin resistance; HOMA-IR) and higher insulin sensitivity (quantitative insulin sensitivity check index; QUICKI) were observed in the monkeys consuming reduced protein formulas (Figures 3, 5). These improvements in glucose metabolism were further coupled with a significant reduction of circulating BCAAs at all time points measured (tested on both 3-way ANCOVA and ANOVA model, *p* < 0.05 for both NMR and AAA data, SI Figure 8). However, other serum markers specific for formula-feeding including methionine, threonine, asparagine, and lysine were not significantly reduced by lowering the protein level in formula (SI Figures 9, 10). At 4 weeks of age, indicators of collagen breakdown (hydroxylysine, hydroxyproline, and glycine), serine (precursor of glycine), homocysteine, and ethanolamine were significantly higher in serum from infants consuming the reduced protein formula (2-way ANOVA, *p* < 0.05 after FDR correction, SI Figures 15A–F). A trend toward higher aspartate was also observed in the infants consuming reduced protein formula (2-way ANOVA, *p* < 0.05 without FDR correction, SI Figure 15G).

As expected, reducing the protein level in infant formula led to a significant change in urinary metabolites, particularly in the first month when physiological and hormonal changes were most profound. In comparison to infants consuming regular formula, those consuming the reduced protein formula exhibited lower levels of urinary amino acids (both essential and non-essential), intermediate products of amino acid metabolism, nitrogenous waste products, and products from microbial degradation of protein or host-microbe co-metabolism (2-way ANOVAs, *p* < 0.05 after FDR correction, SI Figure 16).

### Adding Free Amino Acids in Infant Formula Did Not Alter Milk Intake, but Did Result in Several Metabolic Changes

Addition of free amino acids (such as glutamate) to infant formula has been reported to promote satiation and satiety as well as reduce total intake volume (12). To evaluate the potential role of free amino acids on appetite regulation using the infant rhesus macaque model, four free amino acids present in relatively high concentration in human breast milk (glutamate, glutamine, alanine, and taurine) were added to the formula. We failed to observe an effect of free amino acids on intake and weight gain. Among the four free amino acids added to the formula (glutamate, glutamine, alanine, and taurine), only taurine was observed to be significantly higher in the urine (repeated measures ANOVA, *p* < 0.05 after FDR correction, SI Figure 17). This is expected since unlike most amino acids that have renal reabsorption rates of 98–99%, renal reabsorption of taurine depends on taurine intake, and ranges from 40 to 99.5% (25). Interestingly, for the rhesus infants who consumed formula with added free amino acids, significantly lower levels of pancreatic polypeptide (*p* < 0.05 after FDR correction, SI Figure 4) and a trend toward higher circulating levels of ALK phosphate (*p* < 0.05 before FDR, SI Figure 5) were observed. Addition of free amino acids to formula resulted in a trend toward lower serum levels of betaine (SI Figure 17). Furthermore, several amino acids and nitrogen-containing metabolites were significantly higher in the urine of those receiving free amino acids in their formula (repeated measures ANOVA, *p* < 0.05 after FDR correction, SI Figure 17).

### An Additive Effect of Free Amino Acids With the Base Formula Was Observed

Compared to the infants consuming the reduced protein formula, those consuming the reduced protein formula with added free amino acids revealed the lowest circulating BCAA level (SI Figure 8) and lowest urinary 3-hydroxyisovalerate (byproduct of BCAA degradation, SI Figure 16) in the first month of age, approaching the level observed in the BF reference group.

### Reducing Protein Content in Formula Alters Fecal Microbial Composition in an Intake-Dependent Manner

To evaluate the impact on gut microbial composition, fecal samples collected from the second week onward were examined using 16s rRNA gene sequence analysis. The overall community profile of both BF and FF rhesus infants included the dominant phylum-level representatives from Firmicutes and Actinobacteria, followed by Bacteroides, with the most prevalent bacterial genera identified as *Lactobacillus, Bifidobacteria* and *Prevotella* (SI Figure 18). Differences in fecal microbiota depending on feeding mode (breast or formula) were observed (significantly different OTUs at the genus level are summarized in SI Table 2, evaluated using ANCOM, *p* < 0.05 after FDR correction). However, the fecal microbiome profile of BF rhesus infants is different from that of BF human infants, who tend to be dominated by *Bifidobacteria*. Therefore, only the bacterial community profiles of FF rhesus infants were compared across all time points. A more distinct difference in fecal microbial profile between infants fed regular formula and those fed reduced protein formula was observed in the first month of age (2 and 4 weeks) which was not obvious in the later months (SI Figure 19). This difference is, in part, due to a significantly higher level of fecal *Bifidobacterium* and lower levels of fecal *Dorea* and *Ruminococcus* (from Ruminococcaceae family) in the stools from the reduced protein formula group compared with stools from the regular formula group (SI Figure 20, evaluated using ANCOM, *p* < 0.05 after FDR correction). Fecal samples obtained from monkeys consuming the regular formula had higher diversity than those consuming the reduced protein formula at 4 weeks of age. This observation was not observed in the later weeks (*p* < 0.05, one-way ANOVA follow by *post-hoc* Tukey HSD test). There was no consistent alteration of specific microbial taxa due to free amino acid supplementation in infant formula, suggesting that the added free amino acids may be rapidly absorbed in the upper gastrointestinal tract.

## Discussion

Infant formula is the closest alternative to human milk when breastfeeding is not feasible or desired. The general goal has been to improve the formulation by matching its nutrient content to human milk as closely as possible. However, infant formula largely lacks free amino acids, bioactive and functional proteins, and is formulated with a higher content of protein than human milk. It has been assumed that when energy intake is adequate, protein is utilized for maintaining the body amino acid pool and is deposited in tissue instead of being used as an energy source. Infant formula with more protein beyond what is required does not provide an advantage to infants, as the high levels of circulating amino acids may put an additional burden on the liver and the renal system to metabolize and excrete the excess nitrogen. When optimizing infant formulas, the primary goal should not be to focus on making it similar to human milk, but to make the performance of FF infants similar to that of BF infants. A system-wide omics approach represents a powerful tool for monitoring metabolic consequences and gut microbial profiles in response to early diet beyond what can be captured using clinical growth outcome measurements, and further provides insight for development of improved infant formulas.

In the present study, we validated that the infant rhesus monkey is a highly translational and robust preclinical model to understand the metabolic differences between BF and FF human infants. In comparison to the BF group, all FF groups (regardless of the type of formula) exhibited accelerated weight gain in combination with distinct differences in fecal microbiota composition, as well as serum and urine metabolic profiles. Reducing the protein content in infant formula substantially reduced formula intake and weight gain, as well as serum BCAAs levels, but did not alter other circulating amino acids, such as methionine, threonine, asparagine, and lysine, which were also higher in FF infants compared with BF infants. In addition, through testing study formulas with protein levels slightly lower than rhesus milk, we observed that these FF rhesus infants showed reduced protein catabolism that can be characterized as lowered circulating levels of urea, ammonia, albumin and creatinine as well as lower excretion of creatinine in urine. However, despite having low protein levels in the study formulas, the typical formula-fed phenotype that includes high circulating insulin and amino acids was not improved. Our results suggest that while the total protein in formula is an important factor that could be modified to improve physiological and metabolic profiles of the FF infant, it is not the only factor that contributes to the FF phenotype. We speculate that a dietary component (other than protein alone) may have a role in maintaining the high levels of insulin and C-peptide in these FF infants. Further studies are needed to trace the downstream amino acid catabolic by-products to determine whether BCAA clearance is sub-optimal.

Reducing the protein level in infant formula revealed that the largest physiological, metabolic and fecal microbial differences occur before and at the first month of age (equivalent to approximately 3 months of age in humans). We expect that protein/amino acids from the diet, if utilized efficiently, should largely be absorbed before moving past the terminal ileum. Different microbial profiles obtained from infants consuming regular or reduced protein formulas were observed primarily during the first month of age. These results suggest that, at least during early infancy, high levels of protein may exceed absorption capacity, and reach the colon to become a source of nutrients and influence the abundance of various gut microbes. We observed that as the protein level in infant formula was reduced, levels of fecal microbes (*Bifidobacterium* and *Ruminococcus* from the Ruminococcaceae family) that are known to have varying ability to utilize complex carbohydrates (26) increased (SI Figure 20). After the first month of age, the response toward different protein content in the formula became less pronounced. This time-dependent change paralleled the difference in intake that was most profound in very early age.

Current views suggest that BF infants are born with an innate ability to regulate food intake in response to internal cues of appetite, regardless of mother's milk supply (27). In contrast, bottle-feeding has been proposed to promote more parental control and less self-regulation than breast-feeding (28). This conclusion is partially due to the fact that FF infants have been shown to consume significantly greater volumes of milk than BF infants (8, 9). Regardless, overfeeding of FF infants (who have an associated higher consumption of protein and energy) is associated with increased growth rate and adiposity in early life (29). Early growth acceleration during infancy has been associated with an increased odds of becoming overweight or obese in adult life (30–32). Thus, regulating intake is one key aspect for preventing early growth acceleration.

It is thought that infants have an innate ability to adjust their intake based on the energy density of their food (33); therefore, an appropriate protein: fat ratio may be key to preventing accelerated weight gain during infancy. Several clinical observations have also suggested that the nutrient composition of the diet may also influence how infants regulate their intake. For example, in a parent-blinded, randomized cohort, Ventura *et al*. observed a significant reduction in feeding volume and meal duration when comparing provision of a formula containing free glutamate to an isocaloric standard formula (12, 34). Formula with low protein quality (i.e., with inadequate essential amino acids) may promote higher consumption, as it was observed that infants increased their consumption when being fed a casein-predominant formula regardless of whether the protein:energy ratio was low (35) or high (36). However, when examining formula with a more balanced amino acid profile (whey-casein ratio: 60:40, 2.2 g protein/100 kcal), the differences in daily consumption and energy were not significant when infants were fed a reduced protein formula (70:30, 1.8 g protein/100 kcal) after adjusting for multiple correction, sex, smoke exposure and maternal education (37).

Use of rhesus monkeys as a model provides direct information on consumption since the amount of formula consumed is responsive to infant cues of hunger and satiety and there is no parental encouragement. By feeding isocaloric formulas, we demonstrated that, in early life, higher protein in infant formula induces greater appetite and greater calorie intake, suggesting a lack of ability to completely self-regulate intake based on meal energy density alone. There is a possibility that the early development of the brain reward system toward food intake is incomplete, and the combination of greater energy and protein intake that leads to attenuated circulating BCAAs, insulin, C-peptide, and accelerated growth may preprogram long-term changes. We speculate that a formula with high protein content may diminish the response to internal cues of satiety. However, this finding is only significant in a time sensitive window and needs to be carefully investigated in a clinical study prior to the introduction of complementary food that displaces the intake of breast milk or formula. In this study, we failed to observe a significant effect of free amino acids on regulating formula intake using the rhesus monkey model. However, pancreatic polypeptide, a gut hormone that was previously found to reduce appetite and food intake in humans (38), was lower in the FF groups with additional free amino acids (SI Figure 4).

One potential limitation of the study is that only female rhesus infants were used in order to reduce the within group variations induced by different sexes. In humans, female infants have been shown to have less appetite, are slightly less responsive to cues of feeding and are more sensitive to internal cues of satiety compared to their male counterparts (39). In addition, the formulas used in the current study had a lower protein level (protein accounts for 8.4% energy in the regular formula, and 7.2% in the reduced protein formula) in comparison to the high protein formula used in the European Childhood Obesity Trial [protein provided 11.7% energy in their high protein formula, and 7.1% energy in the low protein formula (40)]. Furthermore, the formulas used in that study were casein-predominant, in which tryptophan is the limiting amino acid; phenylalanine and tyrosine are high in casein. In comparison to the high protein formula, infants who consumed the low protein formula showed lower levels of circulating phenylalanine and tyrosine but these amino acids were still significantly higher than in the BF reference group. In contrast, infants who consumed the low protein formula showed a significantly lower circulating tryptophan level in comparison to the BF reference group (40). In the present study, since whey-predominant formulas were fed, circulating phenylalanine and tyrosine were not different between BF and FF infants, nor were they influenced by the reduced protein level in formula. Additionally, circulating tryptophan was higher in FF compared to BF infants, and was not influenced by the level of protein in the formula.

It is known that mature rhesus milk is considerably higher in protein (15–20 g/L) than human milk (8–9 g/L) (23) and contains significantly less free amino acids (13). This may lead to differences in dietary protein and free amino acids requirement between human and rhesus infants. Human infants may be less tolerant and more metabolically sensitive to high levels of protein in formula. An infant formula designed for human infants may provide a protein level that is considerably high for human infants but approach the lowest level that is acceptable for rhesus infants. Yet, the rhesus macaque is still a valid research model to evaluate the phenotypic and metabolic gap between breast milk and any given formula. The lesson learned from the comparison between formula groups is robust and can serve as a starting point to unveil the biological mechanism behind the “early protein hypothesis.”

## Conclusion

Our data on a preclinical rhesus infant model concludes that protein in formula is an important factor that can be modified to improve the physiological and metabolic outcomes of FF infants. However, although reducing the protein and adding free amino acids to formula is one step forward, it still insufficient to reverse the FF-specific accelerated growth, and BCAA-induced high insulin phenotype. Further research is warranted to explore other dietary factors that may be responsible for inducing the systematic metabolic manifestation that occurs with formula-feeding.

## Data Availability Statement

The 16s sequencing data is available from European Nucleotide Archive (accession code ERP117320) and Qiita (study ID 12033). The raw data supporting the conclusions of this article will be made available from the corresponding author on request, to any qualified researcher.

## Ethics Statement

The animal study was reviewed and approved by University of California, Davis, Institutional Animal Care and Use Committee.

## Author Contributions

CS had full access to all of the data in the study and takes responsibility for the integrity of the data and accuracy of the data analysis. Study concept and design: BL and CR. Metabolomics analysis: XH and JS-O. Microbiome analysis and statistical analyses: XH. Interpretation of data and drafting of manuscript: XH and CS. Obtained funding: BL, CR, and CS. Editing of manuscript: all authors. All authors listed have made a substantial, direct, and intellectual contribution to the work and approved the final version for publication.

### Conflict of Interest

CR is an employee of Mead Johnson. BL has received research grants from Mead Johnson Nutrition and honoraria for lectures at symposia. The remaining authors declare that the research was conducted in the absence of any commercial or financial relationships that could be construed as a potential conflict of interest.
