# Supplementary material for: The Role of Protein and Free Amino Acids on Intake, Metabolism, and Gut Microbiome: A Comparison Between Breast-Fed and Formula-Fed Rhesus Monkey Infants
Source: Front Pediatr. 2020 Jan 24;7:563. doi: 10.3389/fped.2019.00563 (PMC6993202; doi:10.3389/fped.2019.00563)
Supplement: Supplementary file 1 [file Presentation_1.pdf]

Supplementary Information

The role of protein and free amino acids on intake, metabolism and gut microbiome: a comparison between breast-fed and formula-fed rhesus monkey infants

Xuan He[1], Jennie Sotelo-Orozco[1], Colin Rudolph[2], Bo Lönnerdal[1], Carolyn M Slupsky[1,3*]

[1]Department of Nutrition, University of California Davis, CA, United States, [3]Mead Johnson Nutrition, Evansville, IN, United States, 47712, [3]Department of Food Science and Technology, University of California Davis, CA, United States

Table of contents

- Supplementary Tables
  - **SI Table 1.** Nutrient composition of each dietary treatment.
  - **SI Table 2**. Differentiating OTUs between the breast-fed and the formula-fed rhesus infants.
- Supplementary Figures
  - **SI Figure 1.** Comparison of serum urea, blood urea nitrogen (BUN) and ammonia between breast-fed and formula-fed rhesus infants.
  - **SI Figure 2.** Comparison of serum creatinine between breast-fed and formula-fed rhesus infants.
  - **SI Figure 3.** Comparison of urinary creatinine level between breast-fed and formula-fed rhesus infants from the current study and from the previous work.
  - **SI Figure 4**. Comparison of serum pancreatic polypeptide between breast-fed and formula-fed rhesus infants.

- **SI Figure 5.** Comparison of serum total carbon dioxide (TCO2), anion gap, albumin, alanine aminotransferase (ALT), aspartate aminotransferase (AST), alkaline phosphatase (ALK PHOS) and chloride between breast-fed and formula-fed rhesus infants.
- **SI Figure 6.** Comparison of serum glucose, galactose and *myo*-inositol between breast-fed and formula-fed rhesus infants.
- **SI Figure 7.** Comparison of serum hemoglobin, hematocrit and mean corpuscular hemoglobin concentration (MCHC) between breast-fed and formula-fed rhesus infants.
- **SI Figure 8.** Comparison of serum branched chain amino acids (isoleucine, leucine, valine) between breast-fed and formula-fed rhesus infants.
- **SI Figure 9.** Comparison of serum lysine, methionine and threonine between breast-fed and formula-fed rhesus infants.
- **SI Figure 10.** Comparison of serum alanine, asparagine and hydroxyproline between breast-fed and formula-fed rhesus infants.
- **SI Figure 11.** Comparison of serum fumarate, lactate, malate, pyruvate and succinate between breast-fed and formula-fed rhesus infants.
- **SI Figure 12.** Comparison of serum ketone bodies (acetoacetate and 3-hydroxybutyrate) between breast-fed and formula-fed rhesus infants.
- **SI Figure 13.** Serum Phenylalanine and serine concentration that were not statistically significant between the formula-fed and breast-fed rhesus infants in the present study but significantly different in our previous work.
- **SI Figure 14.** The fold change (%) of average daily protein intake from consuming the reduced protein formula.

- **SI Figure 15.** Serum metabolites at 4 weeks of age that are higher in the formula-fed rhesus infants who consumed reduced protein formulas in comparison to those who consumed the regualr formulas.
- **SI Figure 16.** Boxplots of urine metabolites before 1 month of age.
- **SI Figure 17.** The influence of adding free amino acids to infant formula on metabolism.
- **SI Figure 18.** Relative abundance of bacterial genera in feces of rhesus infants consuming rhesus milk, regular formula, regular formula plus free amino acids, reduced protein formula, or reduced protein formula plus free amino acids from 2 to 16 weeks of age.
- **SI Figure 19.** Principal coordinate analysis of weighted and log transformed unifrac distances (A) over time. (B) Shifting of centroids over time.
- **SI Figure 20.** Relative abundance of *Bifidobacterium*, *Dorea* and *Ruminococcus* (from Ruminococcaceae family) from data collected at and before 1 month of age (2 weeks and 4 weeks).

- **References**

SI Table 1. Nutrient composition of each dietary treatment.

	Regular formula	Regular formula plus free AAs	Reduced protein formula	Reduced protein formula plus free AAs	Reference formula [a]	Rhesus milk reference
Protein (g/L)	14.4	14.6	12.3	12.6	18.4	16.6 ± 2.2 [b]
Fat (g/L)	36.6	36.6	36.5	36.5	36.7	50.7 ± 23.6 [c]<br>47.4 ± 15.7 [d]
Carbohydrate (g/L)	75.2	75.2	77.6	77.6	77.7	78.3 ± 3.1 [e]
Lactose (g/L)						81.6 ± 2.1 [f]
Amino acids (μM)						
Ala	7870	8099	6746	6953	9260	7452.3 ± 372.6 [g]
Arg	2161	2161	1852	1852	3474	4478.8 ± 381.2
Asp/Asn	11353	11353	9731	9731	13883	4569.4 ± 500.8
Cys	2483	2483	2129	2129	1526	1643.6 ± 410.9
Glu/Gln	18877	20360	16180	17677	25222	21620.2 ± 566.0
Gly	3836	3836	3288	3288	4886	3094.5 ± 221.0
His	2071	2071	1775	1775	2837	2139.2 ± 213.9
Ile	6746	6746	5783	5783	9089	7211.9 ± 379.6
Leu	12144	12144	10409	10409	15100	14044.2 ± 379.6
Lys	8382	8382	7184	7184	11684	8175.1 ± 681.3
Met	2126	2126	1822	1822	3319	2781.5 ± 222.5
Phe	3358	3358	2879	2879	4774	4421.3 ± 100.5
Pro	9105	9105	7805	7805	13859	16152.9 ± 576.9
Ser	7514	7514	6440	6440	9952	7581.4 ± 473.8
Thr	7886	7886	6760	6760	9700	5575.1 ± 278.8
Trp	1247	1247	1068	1068	1527	ND
Tyr	2359	2359	2022	2022	4454	3756.1 ± 91.6
Val	7670	7670	6574	6574	10192	7371.5 ± 283.5

[a] Values for the reference formula is adapted from (1)

[b] Value is adapted from the true protein measurement of mature rhesus milk (2). True protein was estimated using (nitrogen in whole milk – non-protein nitrogen) X 6.25. Nitrogen was estimated by micro-Kjeldahl analysis.

[c] Value is adapted from measurement of rhesus milk collected after 36 days of lactation (3). Total lipid was measured by a colorimetric method through sulfo-phospho-vanillin reaction.

[d] Value is adapted from measurement of milk collected at 1 month of lactation (4). Total lipid was measured by a micromodification of Rose-Gottleib procedure.

[e] Value is adapted from measurement of milk collected at 1 month of lactation (4). Total carbohydrate was determined by phenol-sulfuric acid method.

[f] Value is adapted from measurement of milk collected after 36 days of lactation (3). Lactose was measured using an enzymatic approach involveing lactase and glucose oxidase.

[g] The amino acid composition was converted using measurements adapted from (5) and corrected using true protein measurement adapted from (2). Amino acid concentration was measured spectrophotometrically after HCl digestion. However, tryptophan was destroyed by acid hydrolysis therefore not included in the total amino acid concentration. Due to this limitation of excluding tryptophan as a part of total amino acid, the concentration for each amino acid present in the table is over-estimated.

Data on rhesus milk are presented from literature as Mean ± SD. For unit conversion, rhesus milk density is conventionally assumed as 1.03 g/mL. ND: not determined.

SI Table 2. Differentiating OTUs between the breast-fed and the formula-fed rhesus infants. ● High in breast-fed, ○ High in the formula-fed. Significance is evaluated using Analysis of Composition of Microbiomes (ANCOM) with $p < 0.05$ after FDR correction.

**Taxa (Phylum)**	**Taxa (family genus)**	**Age (weeks)**							
		**2**	**4**	**6**	**8**	**10**	**12**	**14**	**16**
Actinobacterium	*Corynebacteriaceae Corynebacterium*			●	●		●		●
Firmicutes	*Lactobacillaceae Lactobacillus*			●	●		●		
Firmicutes	*Lachnospiraceae Lachnospira*				●		●	●	●
Firmicutes	*Clostridiaceae SMB53*			●		●			
Firmictues	*Streptococcaceae Lactococcus*		○	○	○			○	○
Firmicutes	*Veillonellaceae Anaerovibrio*	○	○		○	○	○	○	○
Bacteroidetes	*Paraprevotellaceae Prevotella*			○		○			○
Firmicutes	*Veillonellaceae Megasphaera*			○		○	○	○	

SI Figure 1. Comparison of serum urea, blood urea nitrogen (BUN) and ammonia between breast-fed (black) and formula-fed rhesus infants (red, orange, green, blue). Serum urea, BUN and ammonia were quantified using NMR, biochemical assay and AAA, respectively. Data are presented as mean ± SEM.

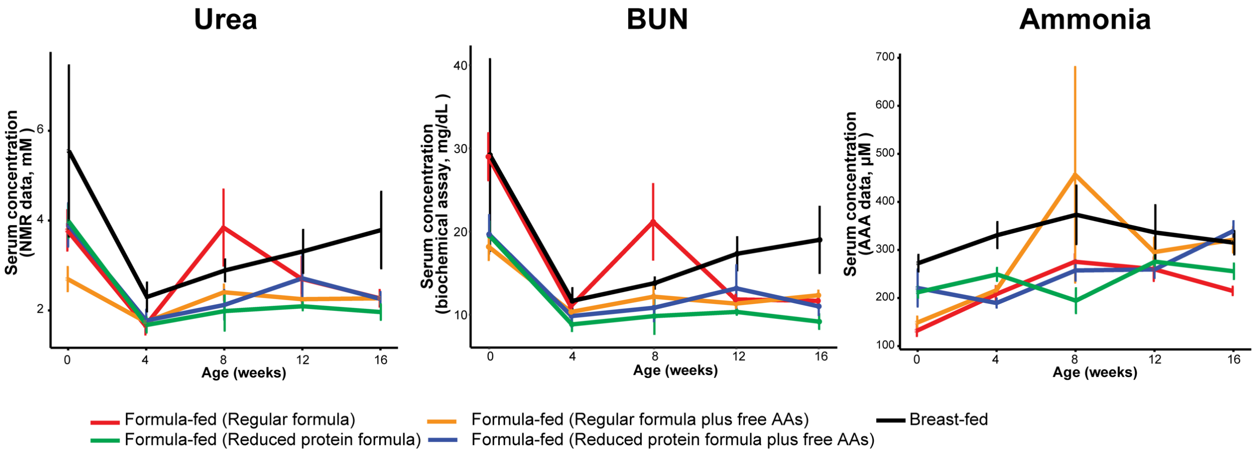

SI Figure 2. Comparison of serum creatinine between breast-fed (black) and formula-fed rhesus infants (red, orange, green, blue). Data were quantified using NMR (left) and biochemical assay (right). Data are presented as mean ± SEM.

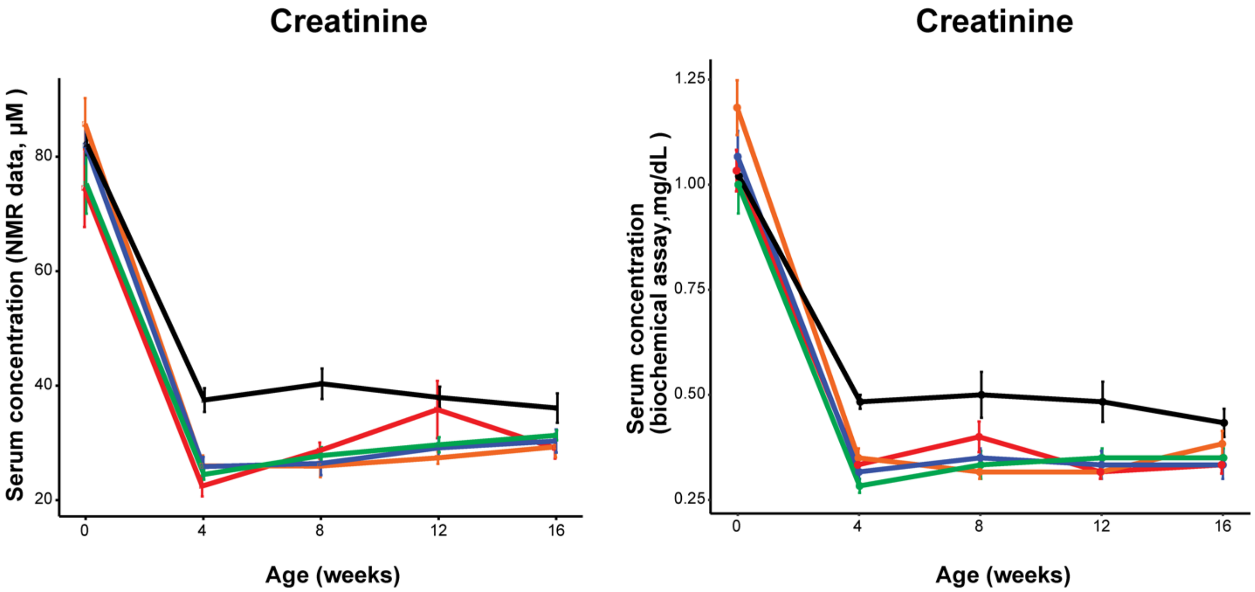

SI Figure 3. Comparison of urinary creatinine level between breast-fed (BF) and formula-fed (FF) rhesus infants from the current study and from the previous work ((1), BF ref, FF ref). Data are presented as mean ± SEM.

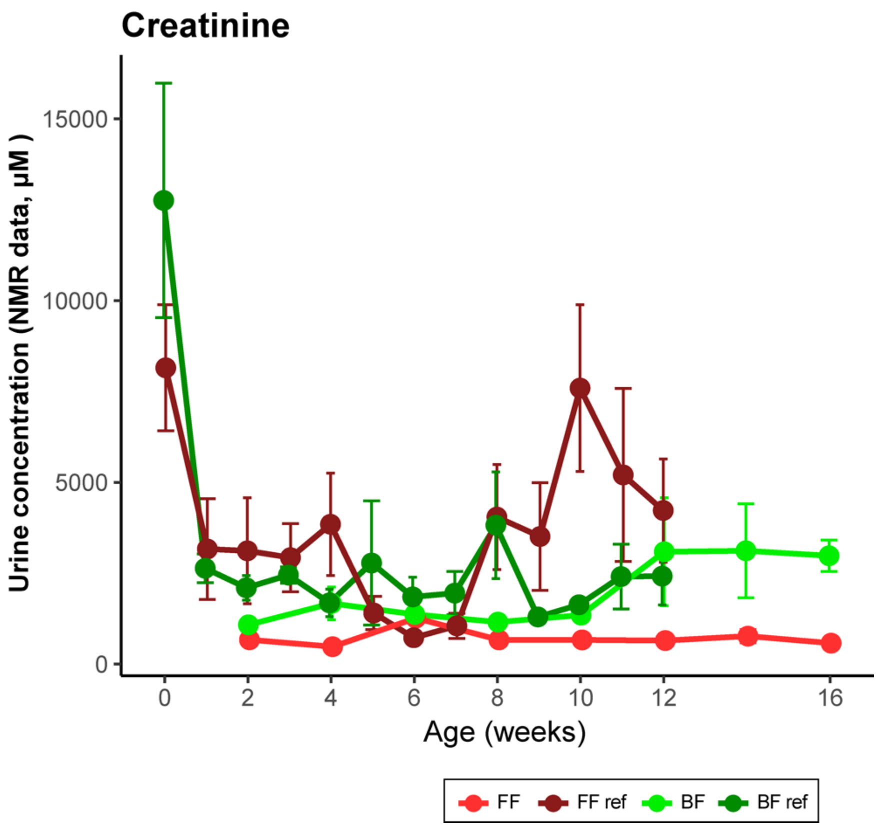

SI Figure 4. Comparison of serum pancreatic polypeptide between breast-fed (black) and formula-fed rhesus infants (red, orange, green, blue). Data are presented as mean ± SEM.

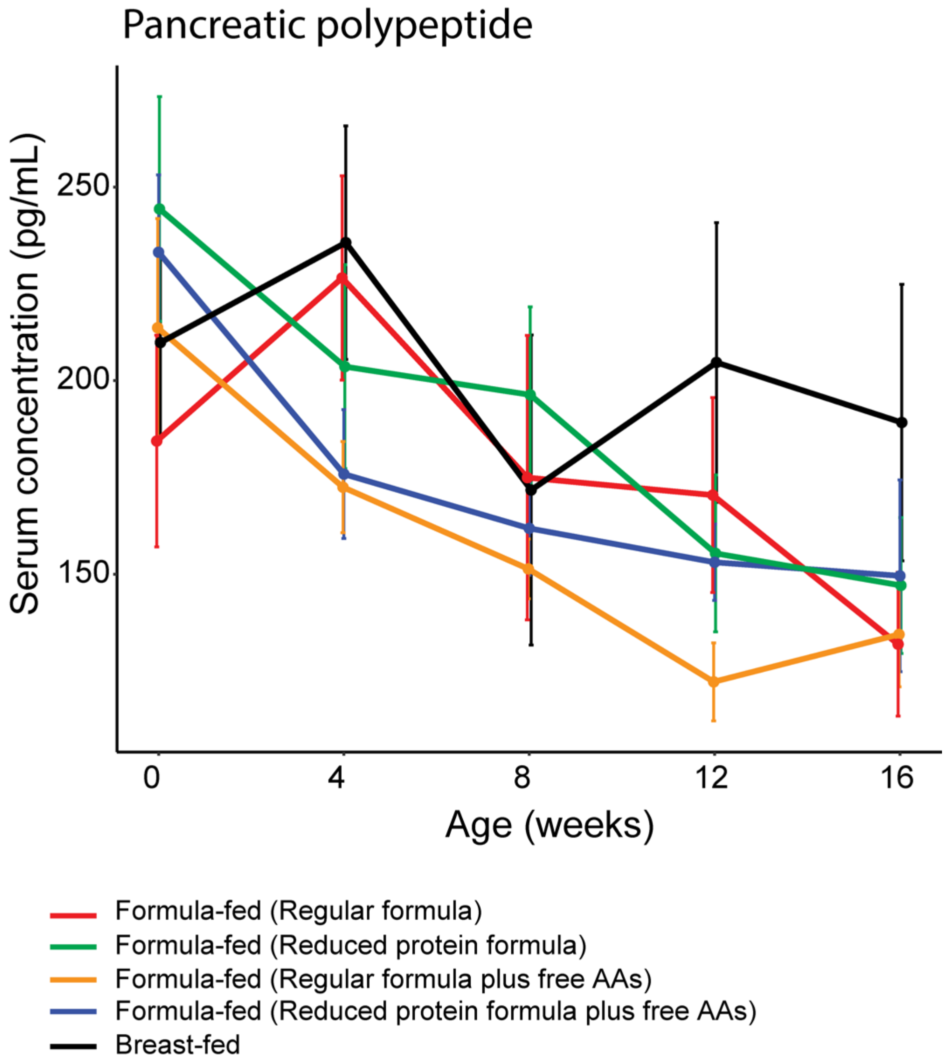

SI Figure 5. Comparison of serum total carbon dioxide (TCO2), anion gap, albumin, alanine aminotransferase (ALT), aspartate aminotransferase (AST), alkaline phosphatase (ALK PHOS) and chloride between breast-fed (black) and formula-fed rhesus infants (red, orange, green, blue). Data are presented as mean ± SEM.

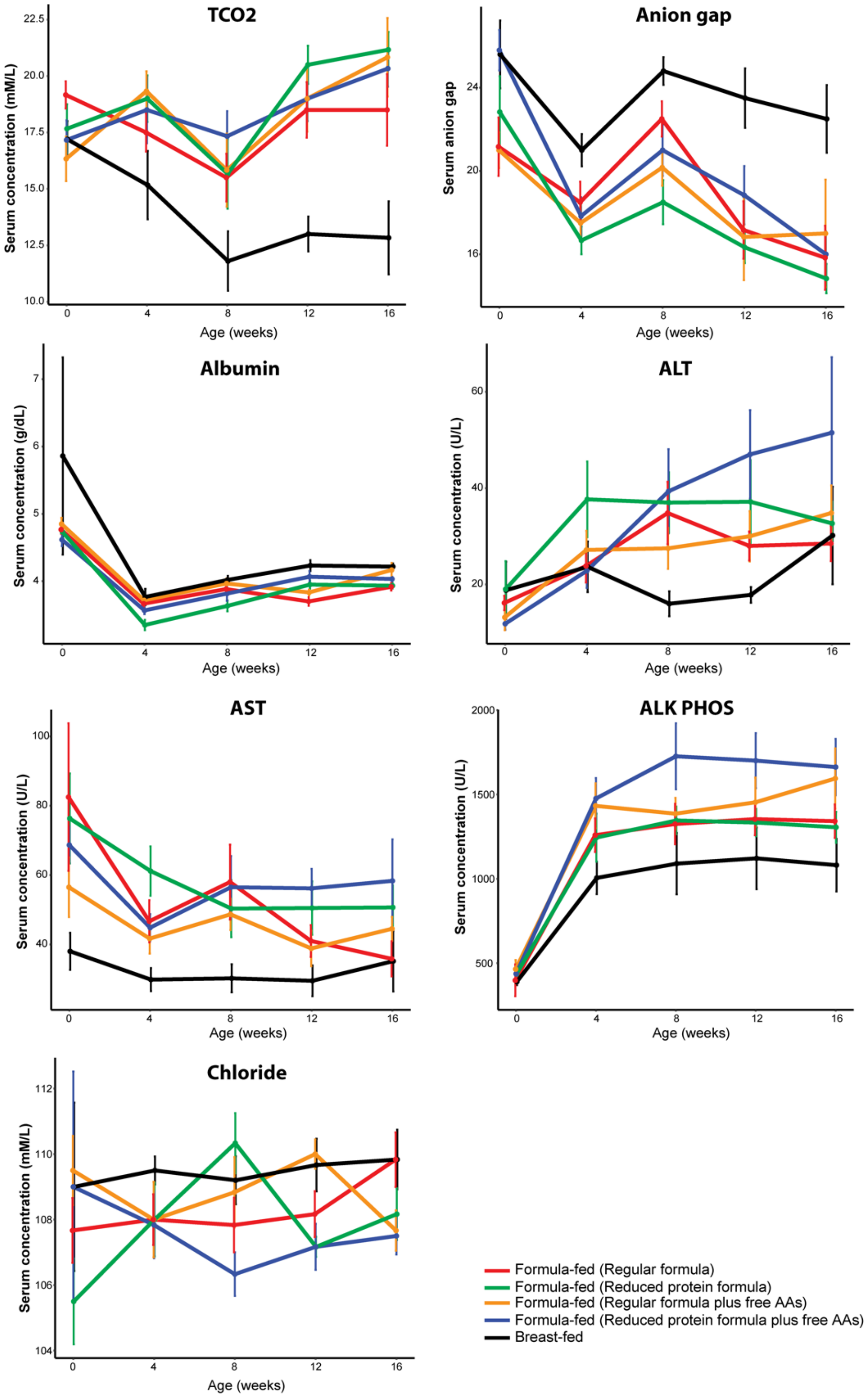

SI Figure 6. Comparison of serum glucose, galactose and *myo*-inositol between breast-fed (black) and formula-fed rhesus infants (red, orange, green, blue). Measurement of glucose was conducted using NMR (top left) and biochemical assay (top right). Data are presented as mean ± SEM.

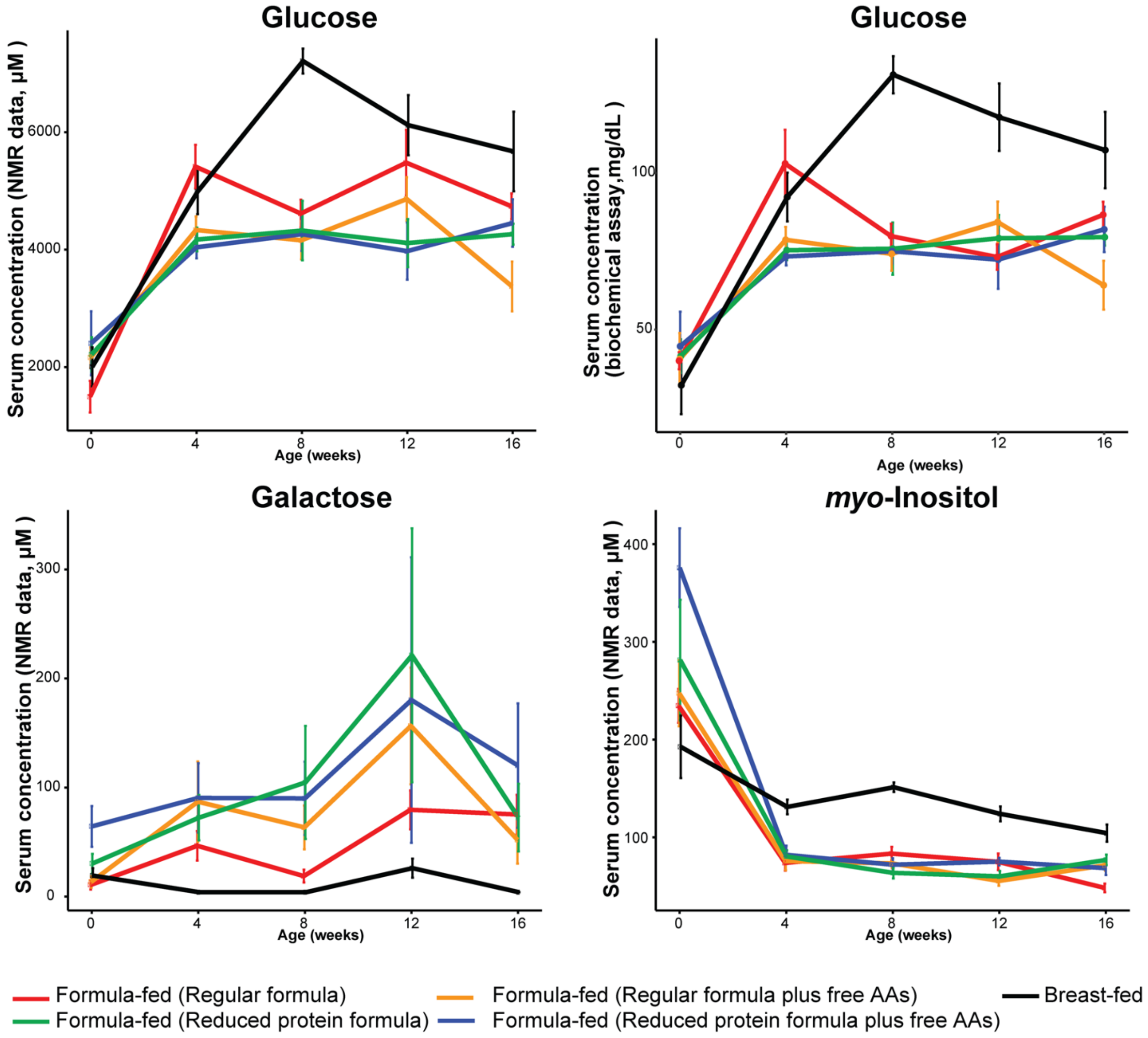

SI Figure 7. Comparison of serum hemoglobin, hematocrit and mean corpuscular hemoglobin concentration (MCHC) between breast-fed (black) and formula-fed rhesus infants (red, orange, green, blue). Data are presented as mean ± SEM.

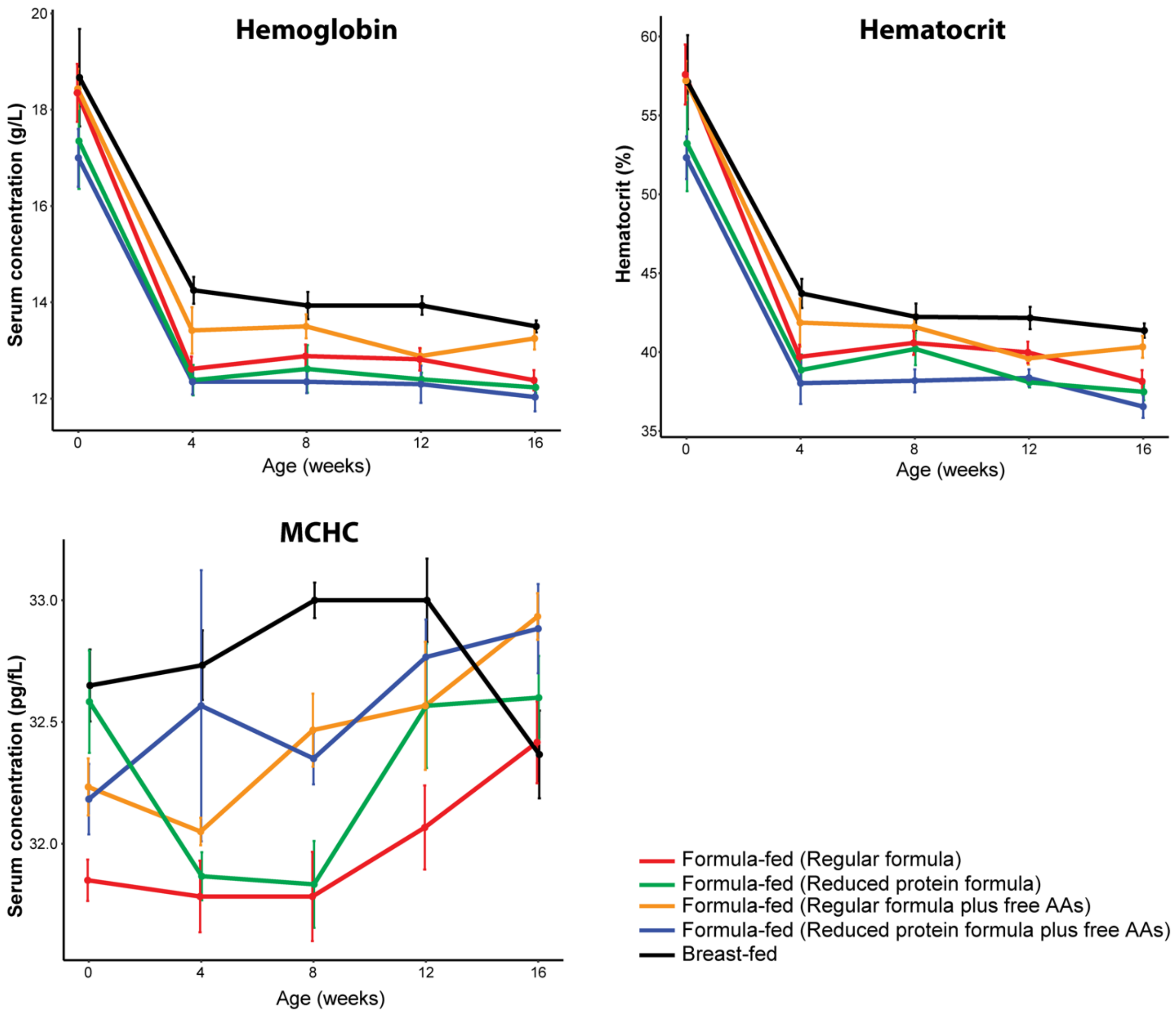

SI Figure 8. Comparison of serum branched chain amino acids (isoleucine, leucine, valine) between breast-fed (black) and formula-fed rhesus infants (red, orange, green, blue). Data were quantified using NMR (left) and AAA (right). Data are presented as mean ± SEM.

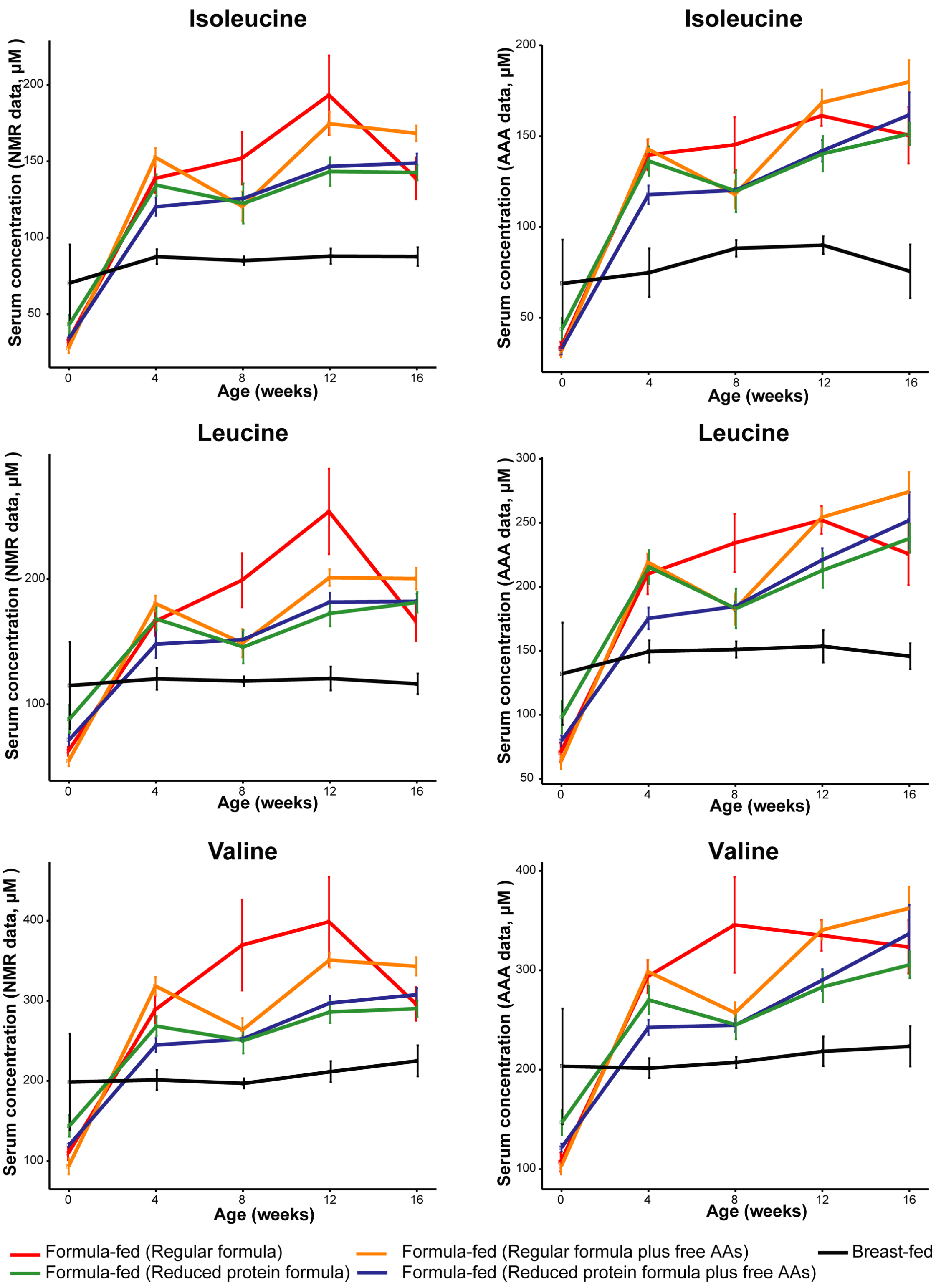

SI Figure 9. Comparison of serum lysine, methionine and threonine between breast-fed (black) and formula-fed rhesus infants (red, orange, green, blue). Data were quantified using NMR (left) and AAA (right). Data are presented as mean ± SEM.

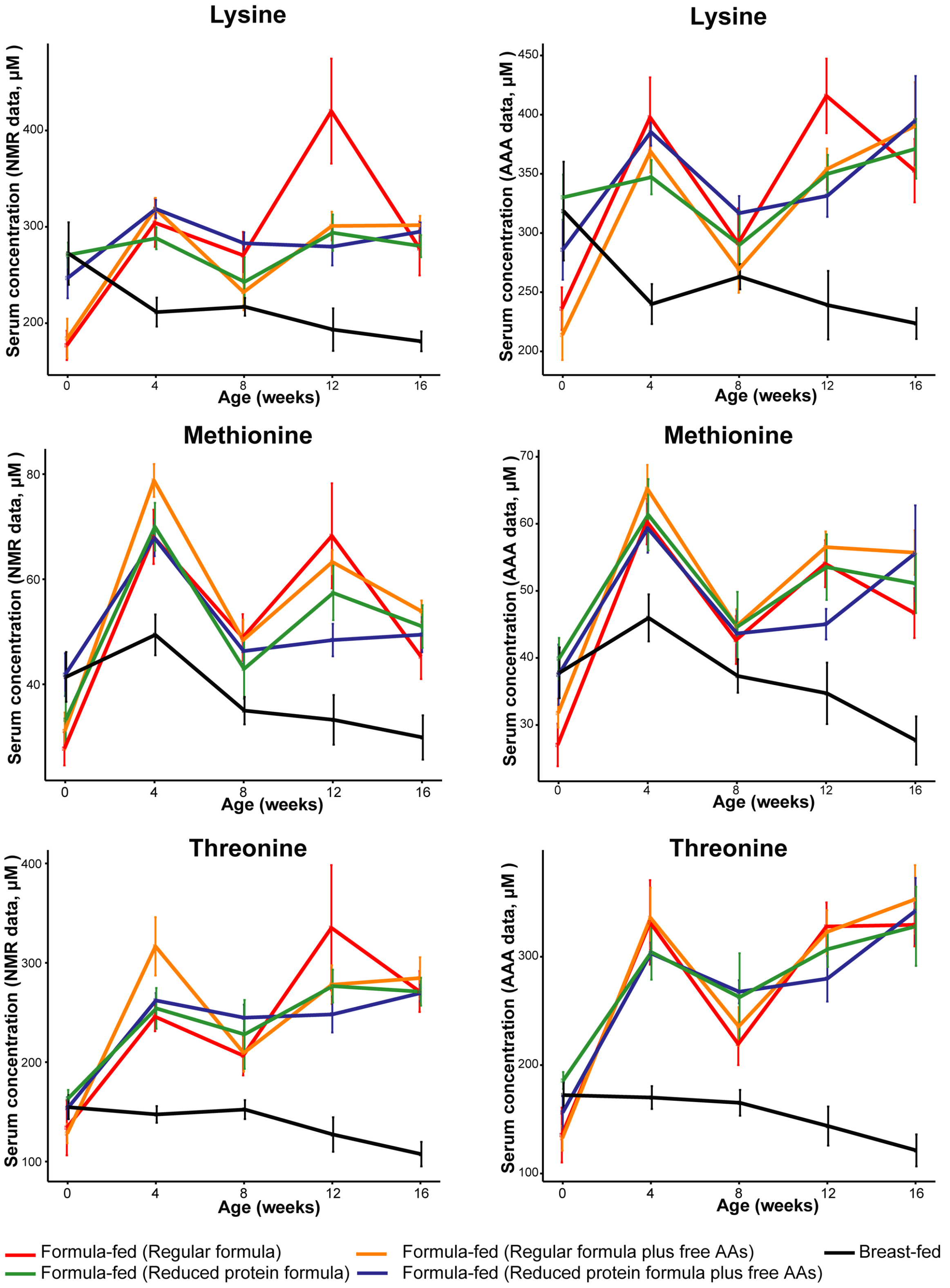

SI Figure 10. Comparison of serum alanine, asparagine and hydroxyproline between breast-fed (black) and formula-fed rhesus infants (red, orange, green, blue). Data were quantified using NMR (left) and AAA (right). Data are presented as mean ± SEM.

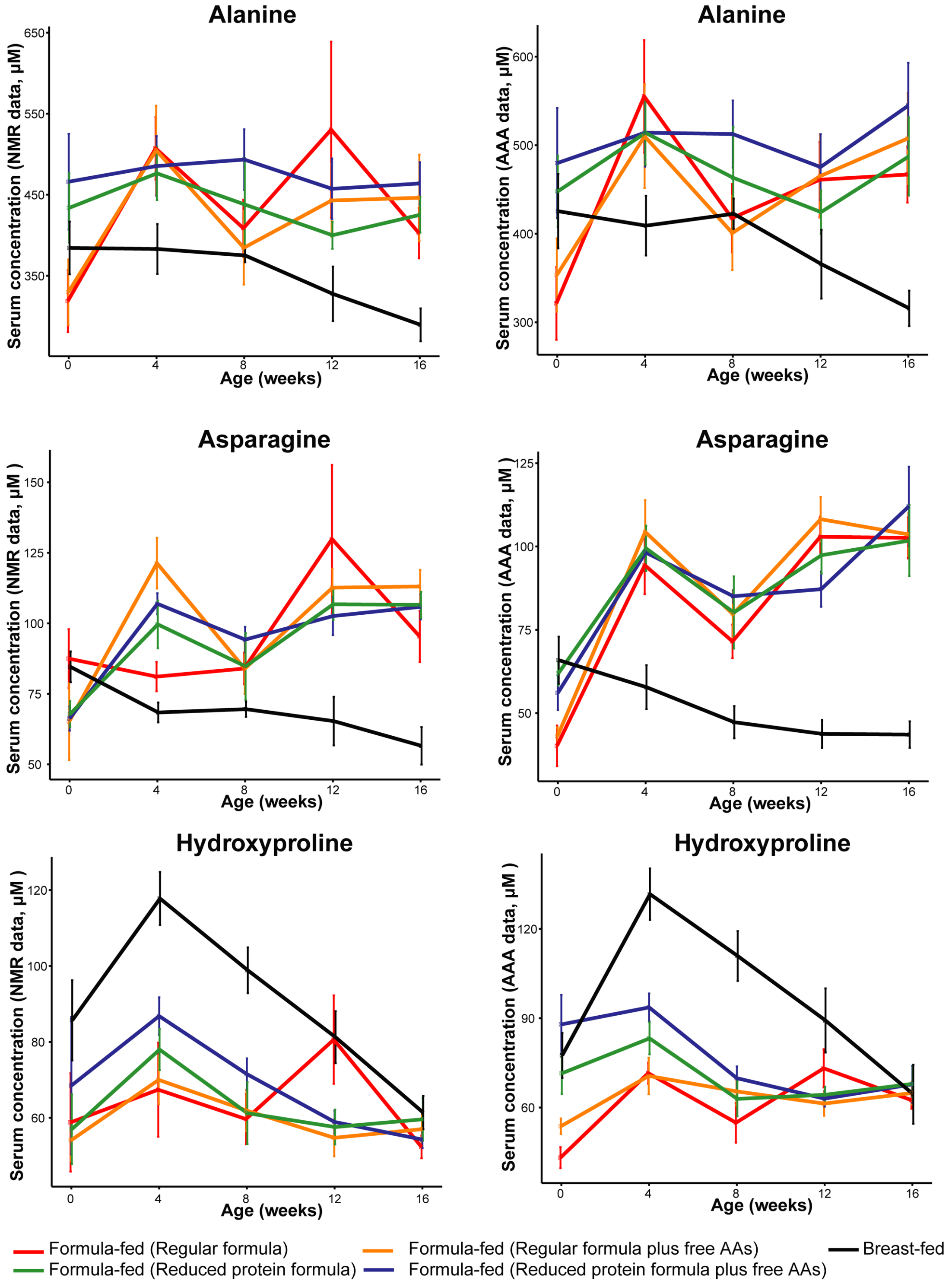

SI Figure 11. Comparison of serum fumarate, lactate, malate, pyruvate and succinate between breast-fed (black) and formula-fed rhesus infants (red, orange, green, blue). Data are presented as mean ± SEM.

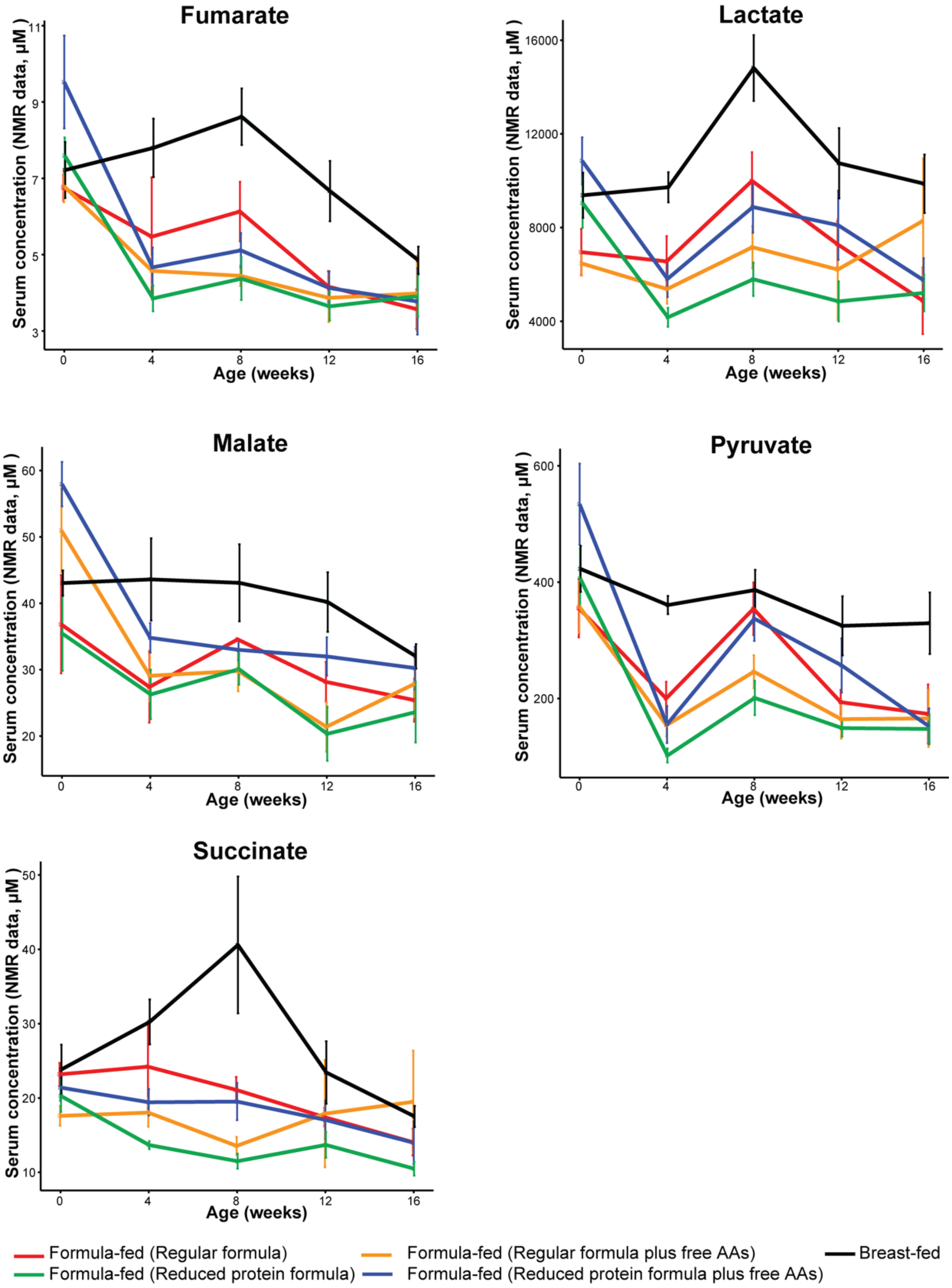

SI Figure 12. Comparison of serum ketone bodies (acetoacetate and 3-hydroxybutyrate) between breast-fed (black) and formula-fed rhesus infants (red, orange, green, blue). Data are presented as mean ± SEM.

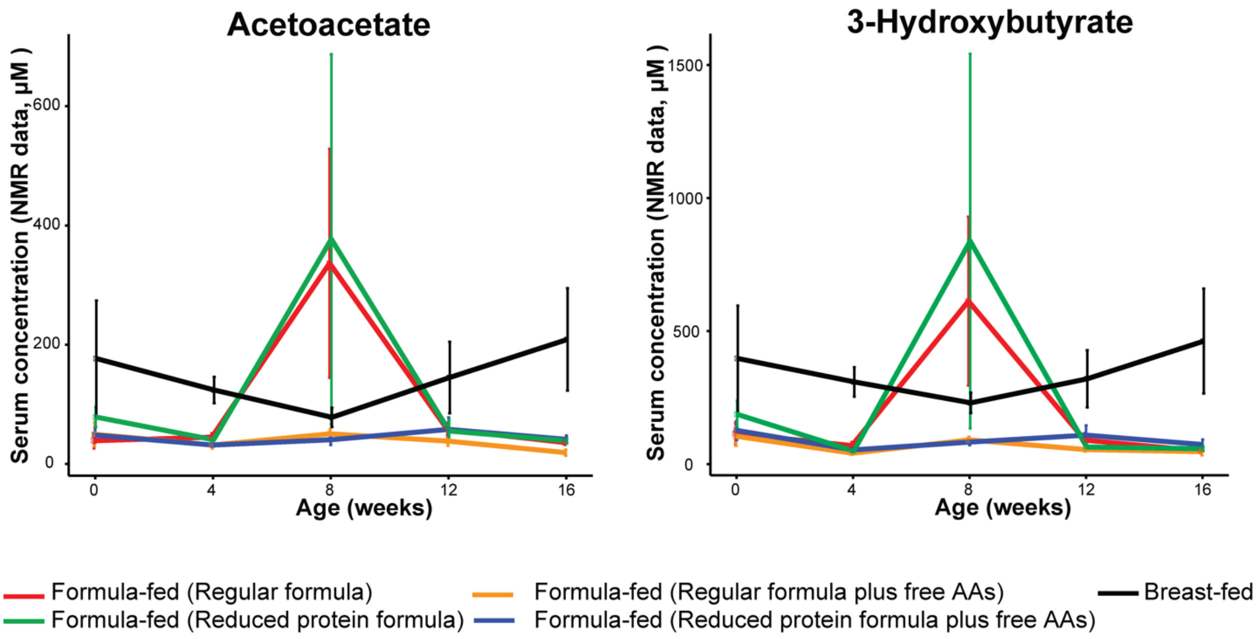

SI Figure 13. Serum Phenylalanine and serine concentration that were not statistically significant between the formula-fed (FF) and breast-fed (BF) rhesus infants in the present study but significantly different in our previous work ((1), BF ref, FF ref). Statistical difference was evaluated using repeated measures ANCOVA. Data are presented as mean ± SEM.

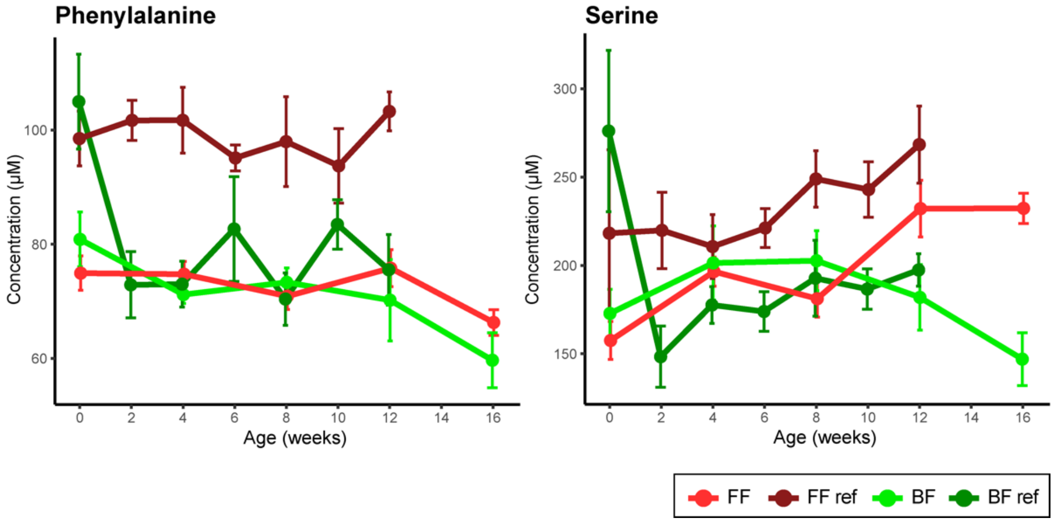

SI Figure 14. The fold change (%) of average daily protein intake from consuming the reduced protein formula. The smooth curve was fitted using loess regression and the 95% confidence interval was constructed using a t-based approximation.

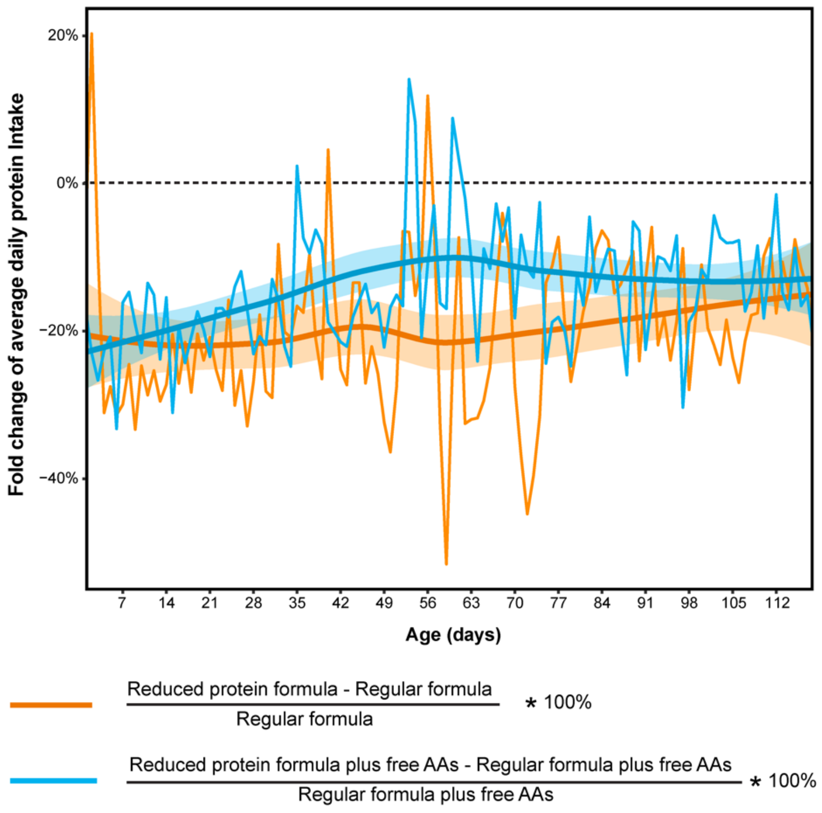

SI Figure 15. Serum metabolites at 4 weeks of age that are higher in the formula-fed rhesus infants who consumed reduced protein formulas in comparison to those who consumed the regualr formulas. (A). 4-hydroxyproline, (B). hydroxylysine, (C). glycine, (D). serine, (E). homocysteine, and (F). ethanolamine were significant ($p<0.05$) after adjustment of multiple comparison using FDR.(G). aspartate shows an increasing trend in those who consumed the reduced protein formulas ($p<0.05$ before FDR correction). Statistical significance was evaluated using multiple 2-way ANOVAs.

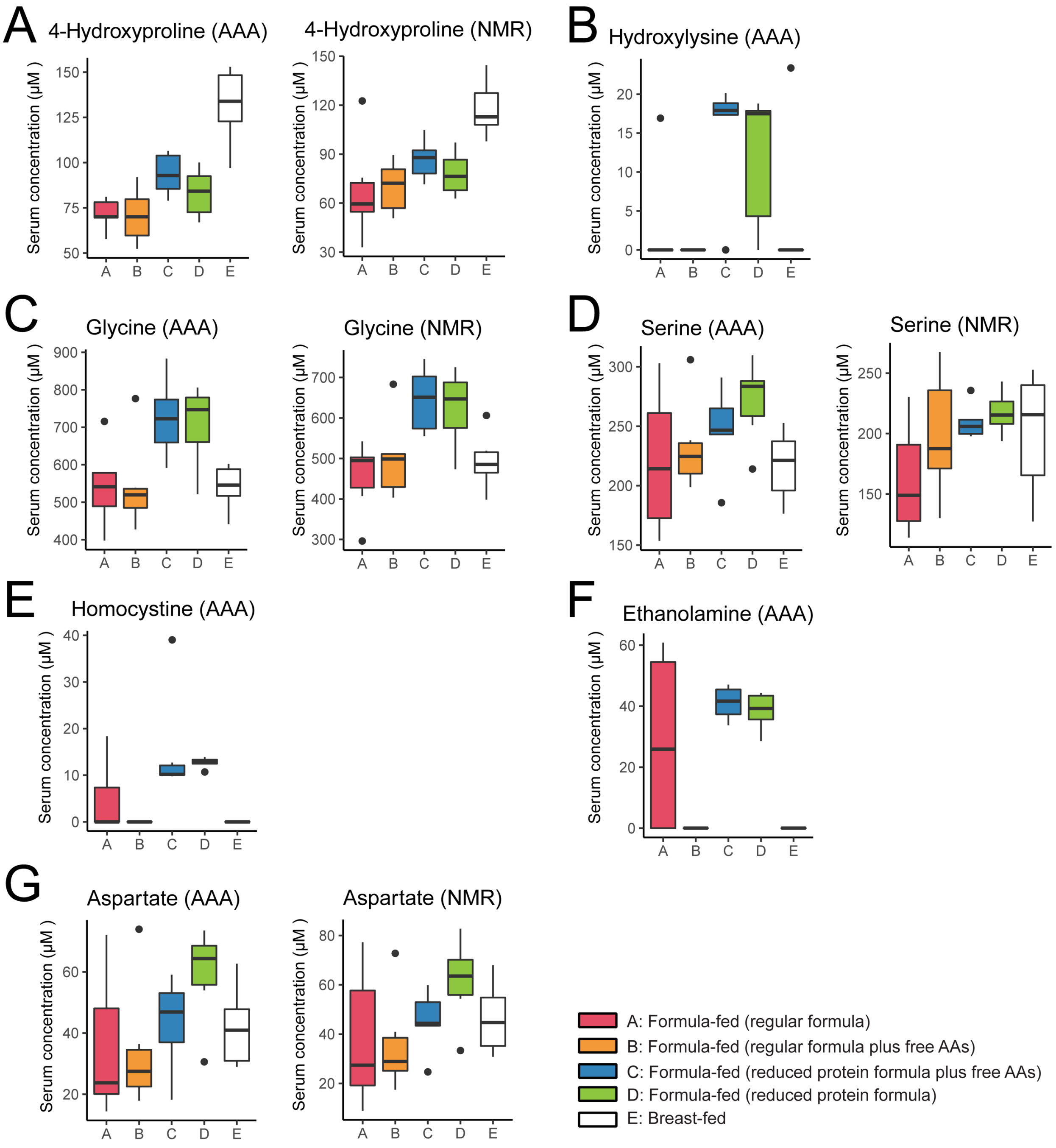

SI Figure 16. Boxplots of urine metabolites before 1 month of age. The data presented here is the combination of measurements at 2 and 4 weeks of age. Essential AAs (lysine, phenylalanine, valine), non-essential AAs (tyrosine, ornithine), AA metabolism intermediate products and nitrogen waste products (3-hydroyisobutyrate, 3-hydroxyisovalerate, allantoin, dimethylglycine, sarcosine, urea), and products from microbial degradation of protein or host-microbe co-metabolism (2-hydroxyisobutyrate, 3-indoxylsulfate, 4-hydroxyphenylacetate, dimethyl sulfone, phenylacetate, phenylacetylglycine) were significantly lower with reduced protein in infant formula (p<0.05 after FDR correction, two-way ANOVA). Urinary level of histidine, threonine, isoleucine and glycine showed a trend toward lower concentrations in reduced protein formula (p<0.05 before FDR correction, two-way ANOVA).

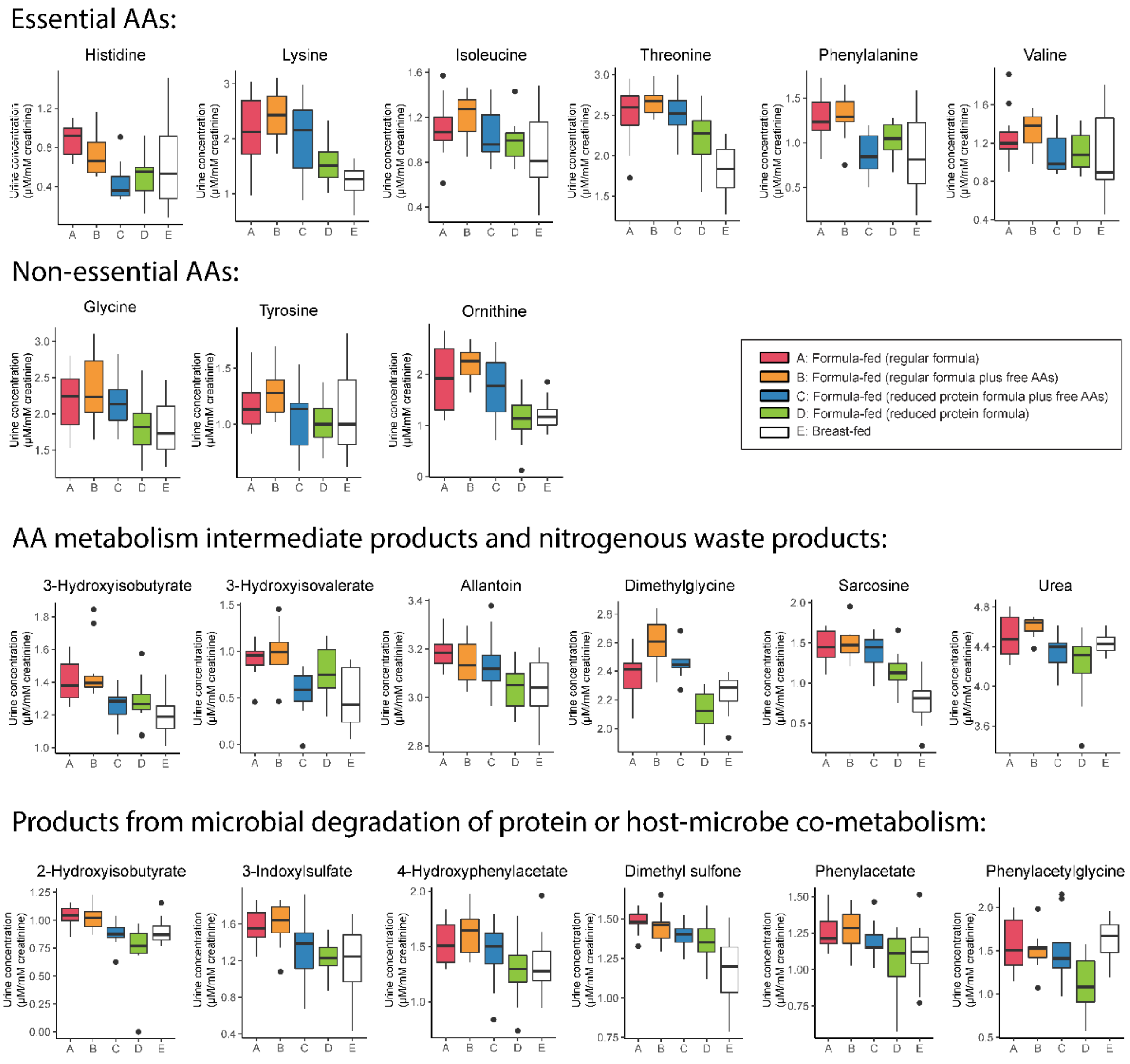

SI Figure 17. The influence of adding free amino acids to infant formula on metabolism. In urine, amino acids (aspartate, proline, threonine), amino acid derivatives (hydroxyproline, dimethylglycine, cadaverine) were significantly higher in the urine of infants fed formulas with the addition of free amino acids ($p<0.05$ after FDR correction, repeated measures ANOVA). Urinary asparagine, betaine, glycine isoleucine and serine showed a tendency to be higher in the urine those infants receiving formula with the addition of free amino acids ($p<0.05$ before FDR correction, repeated measures ANCOVA). Serum betaine showed a trend toward lower levels in infants fed formula with the addition of free amino acids ($p<0.05$ before FDR correction, repeated measures ANOVA). Data are presented as mean ± SEM.

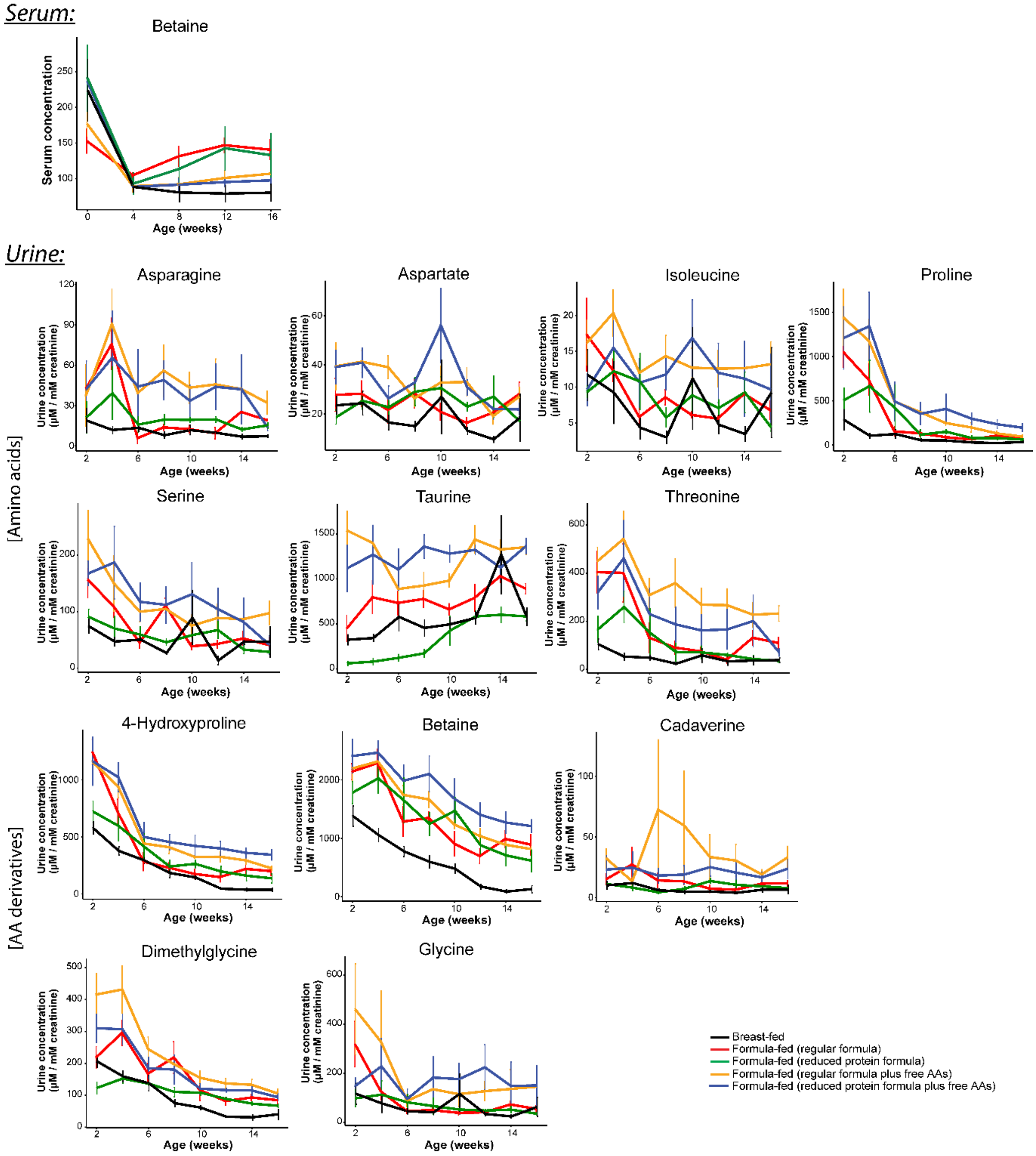

SI Figure 18. Relative abundance of bacterial genera in feces of rhesus infants consuming rhesus milk, regular formula, regular formula plus free amino acids, reduced protein formula, or reduced protein formula plus free amino acids from 2 to 16 weeks of age.

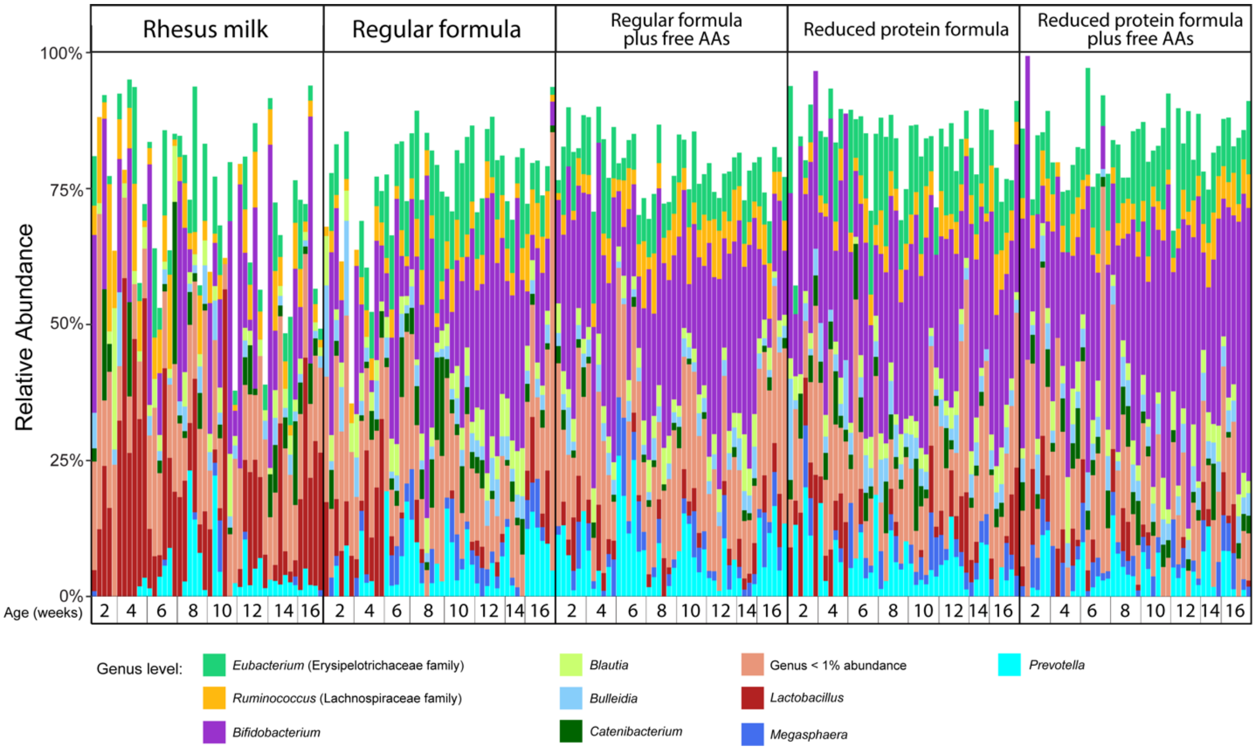

SI Figure 19. Principal coordinate analysis of weighted and log transformed unifrac distances (A) over time. (B) Shifting of centroids over time. The centroids are calculated using the average of PC1 and PC2 within each cluster.

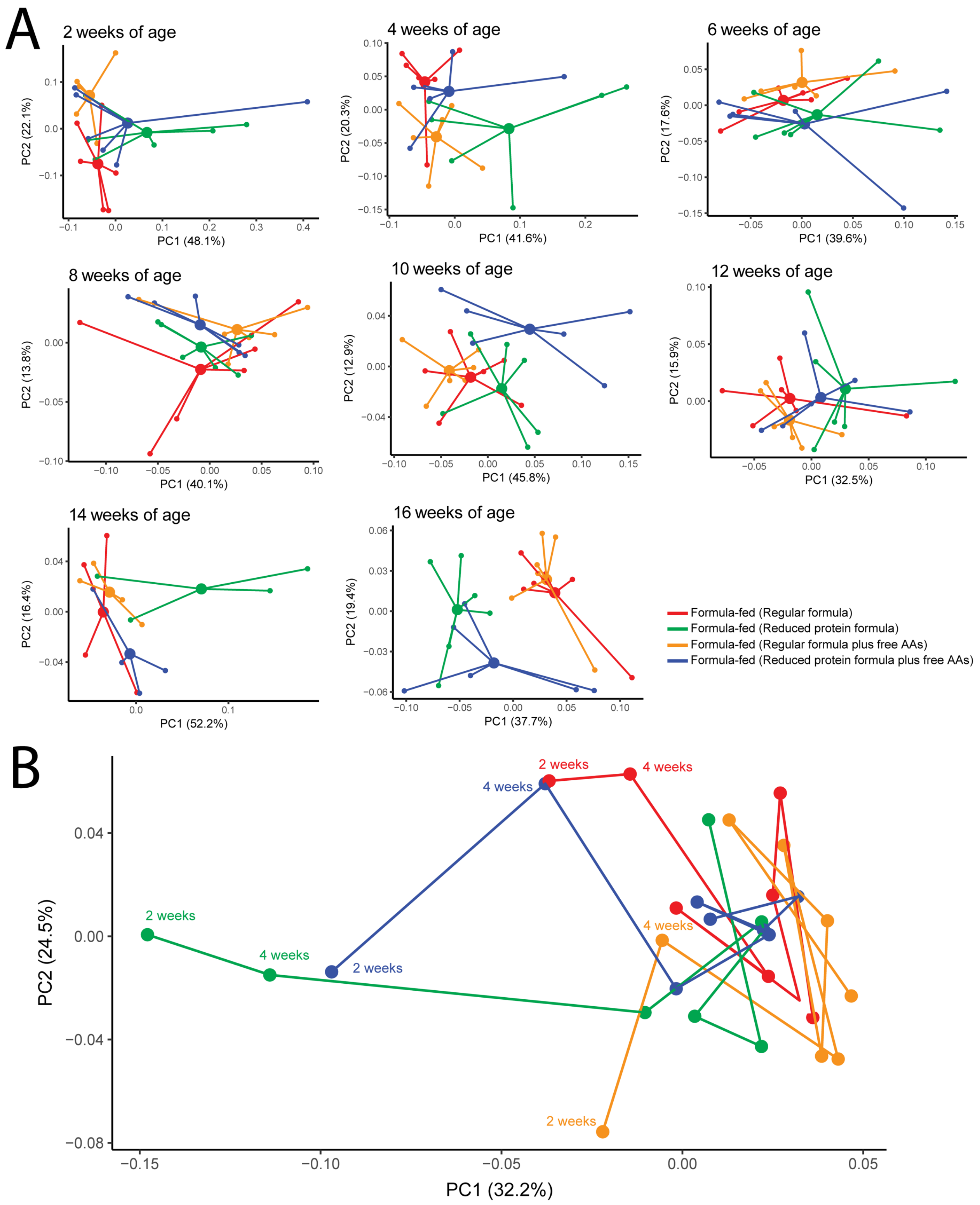

SI Figure 20. Relative abundance of *Bifidobacterium*, *Dorea* and *Ruminococcus* (from Ruminococcaceae family) from data collected at and before 1 month of age (2 weeks and 4 weeks).

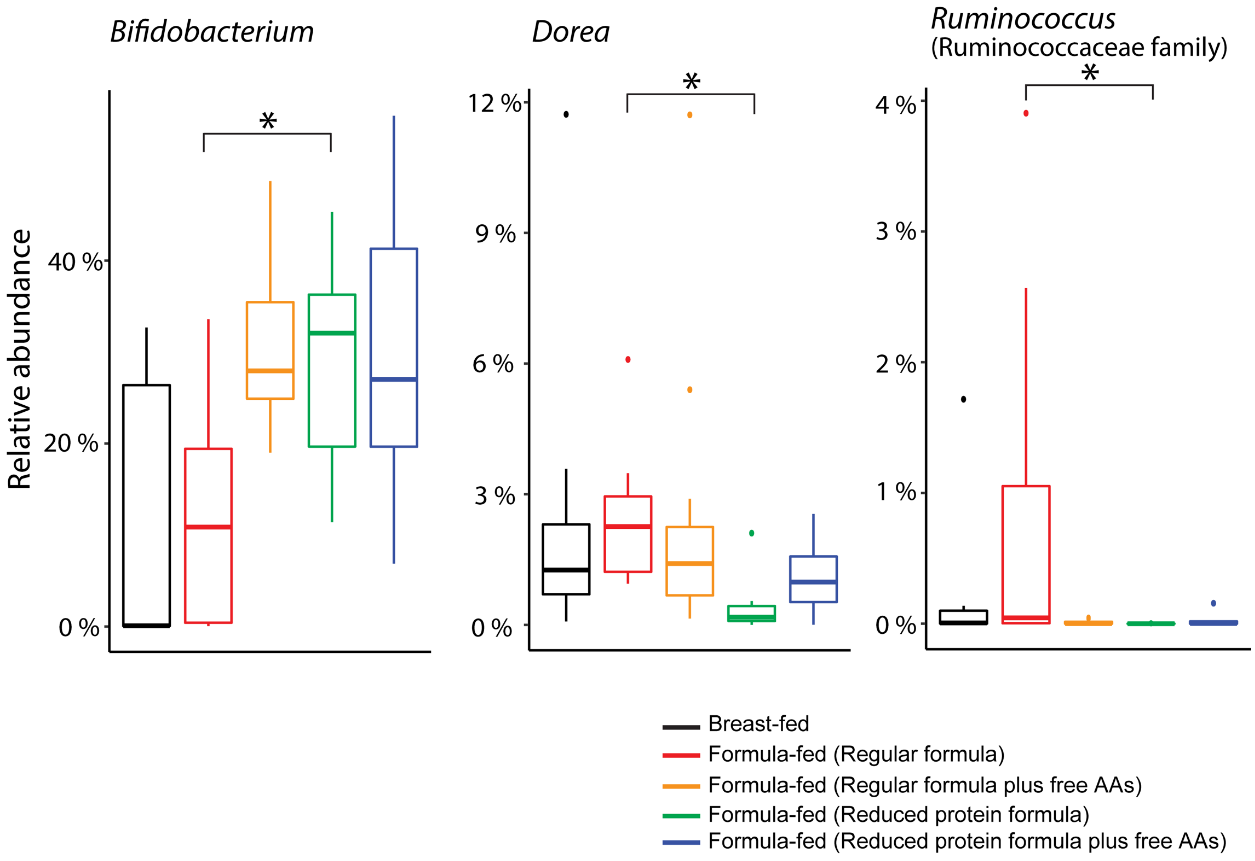

References

1. O'Sullivan A, He X, McNiven EMS, Haggarty NW, Lönnerdal B, Slupsky CM. Early diet impacts infant rhesus gut microbiome, immunity, and metabolism. *J Proteome Res* (2013) **12**:2833–2845. doi:10.1021/pr4001702

2. Kunz C, Lönnerdal B. Protein composition of rhesus monkey milk: comparison to human milk. *Comp Biochem Physiol Comp Physiol* (1993) **104**:793–797.

3. Lönnerdal B, Keen CL, Glazier CE, Anderson J. A longitudinal study of rhesus monkey (Macaca mulatta) milk composition: trace elements, minerals, protein, carbohydrate, and fat. *Pediatr Res* (1984) **18**:911–914. doi:10.1203/00006450-198409000-00023

4. Hinde K, Power ML, Oftedal OT. Rhesus macaque milk: magnitude, sources, and consequences of individual variation over lactation. *Am J Phys Anthropol* (2009) **138**:148–157. doi:10.1002/ajpa.20911

5. Davis TA, Nguyen HV, Garcia-Bravo R, Fiorotto ML, Jackson EM, Lewis DS, Lee DR, Reeds PJ. Amino acid composition of human milk is not unique. *J Nutr* (1994) **124**:1126–1132. doi:10.1093/jn/124.7.1126
